# Diabetes and anti-diabetic interventions and the risk of gynaecological and obstetric morbidity: an umbrella review of the literature

**DOI:** 10.1186/s12916-023-02758-1

**Published:** 2023-04-18

**Authors:** Anita Semertzidou, Harriet Grout-Smith, Ilkka Kalliala, Akanksha Garg, Vasso Terzidou, Julian Marchesi, David MacIntyre, Phillip Bennett, Konstantinos Tsilidis, Maria Kyrgiou

**Affiliations:** 1 Department of Metabolism, Digestion and Reproduction – Surgery and Cancer, Faculty of Medicine, Imperial College London, London, UK; 2 Department of Obstetrics and Gynaecology, University of Helsinki and Helsinki University Hospital, Helsinki, Finland; 3 Queen Charlotte’s and Chelsea – Hammersmith Hospital, Imperial College Healthcare NHS Trust, London, UK; 4 School of Biosciences, Cardiff University, Cardiff, UK; 5 Department of Hygiene and Epidemiology, University of Ioannina School of Medicine, Ioannina, Greece; 6 Department of Epidemiology and Biostatistics, School of Public Health, Imperial College London, London, UK

**Keywords:** Diabetes, Gynaecology, Obstetrics, Cancer, Macrosomia, Prematurity, Large for gestational age, Congenital malformations

## Abstract

**Background:**

Diabetes has reached epidemic proportions in recent years with serious health ramifications. The aim of this study was to evaluate the strength and validity of associations between diabetes and anti-diabetic interventions and the risk of any type of gynaecological or obstetric conditions.

**Methods:**

Design: Umbrella review of systematic reviews and meta-analyses.

Data sources: PubMed, Medline, Embase, Cochrane Database of Systematic Reviews, manual screening of references.

Eligibility criteria: Systematic reviews and meta-analyses of observational and interventional studies investigating the relationship between diabetes and anti-diabetic interventions with gynaecological or obstetric outcomes. Meta-analyses that did not include complete data from individual studies, such as relative risk, 95% confidence intervals, number of cases/controls, or total population were excluded.

Data analysis: The evidence from meta-analyses of observational studies was graded as strong, highly suggestive, suggestive or weak according to criteria comprising the random effects estimate of meta-analyses and their largest study, the number of cases, 95% prediction intervals, *I*^2^ heterogeneity index between studies, excess significance bias, small study effect and sensitivity analysis using credibility ceilings. Interventional meta-analyses of randomised controlled trials were assessed separately based on the statistical significance of reported associations, the risk of bias and quality of evidence (GRADE) of included meta-analyses.

**Results:**

A total of 117 meta-analyses of observational cohort studies and 200 meta-analyses of randomised clinical trials that evaluated 317 outcomes were included. Strong or highly suggestive evidence only supported a positive association between gestational diabetes and caesarean section, large for gestational age babies, major congenital malformations and heart defects and an inverse relationship between metformin use and ovarian cancer incidence. Only a fifth of the randomised controlled trials investigating the effect of anti-diabetic interventions on women’s health reached statistical significance and highlighted metformin as a more effective agent than insulin on risk reduction of adverse obstetric outcomes in both gestational and pre-gestational diabetes.

**Conclusions:**

Gestational diabetes appears to be strongly associated with a high risk of caesarean section and large for gestational age babies. Weaker associations were demonstrated between diabetes and anti-diabetic interventions with other obstetric and gynaecological outcomes.

**Trial registration:**

Open Science Framework (OSF) (Registration https://doi.org/10.17605/OSF.IO/9G6AB).

**Supplementary Information:**

The online version contains supplementary material available at 10.1186/s12916-023-02758-1.

## Background

Diabetes mellitus (DM) affects 223 million women (20–79 years) worldwide according to 2019 estimates, a number which is expected to rise to 343 million by 2045 [1]. According to the International Diabetes Federation (IDF), 1 in 6 pregnancies is affected by diabetes; 13.6% of cases represent pregestational diabetes (PGDM), while 86.4% represent gestational diabetes mellitus (GDM) [1]. In the UK, approximately 16 out of every 100 women will develop gestational diabetes [2]. Several risk factors, such as ethnicity, age, family history, smoking and high blood pressure have been acknowledged as contributing to disease development, but the epidemic proportions noted over the last few decades are attributable to obesity and physical inactivity [2].

The epidemiological burden of diabetes in women is accompanied by a wide spectrum of adverse gynaecological and obstetric outcomes. Reports have associated diabetes with endometrial and ovarian carcinogenesis affecting both incidence and mortality [3–7], and there exists a well described relationship between diabetes, either pre-existing or gestational, and obstetric morbidity [8–18].

Umbrella reviews aim to critically assess the totality of evidence provided by systematic reviews and meta-analyses and test the validity of reported estimates. In this study, we performed an umbrella review of systematic reviews and meta-analyses on the association between diabetes and anti-diabetic interventions, including drug, dietary and lifestyle interventions, and the risk of any type of gynaecological or obstetric morbidity with the aim to evaluate the strength of available evidence and guide future healthcare policies.

## Methods

### Search strategy and selection criteria

**Fig. 1:**
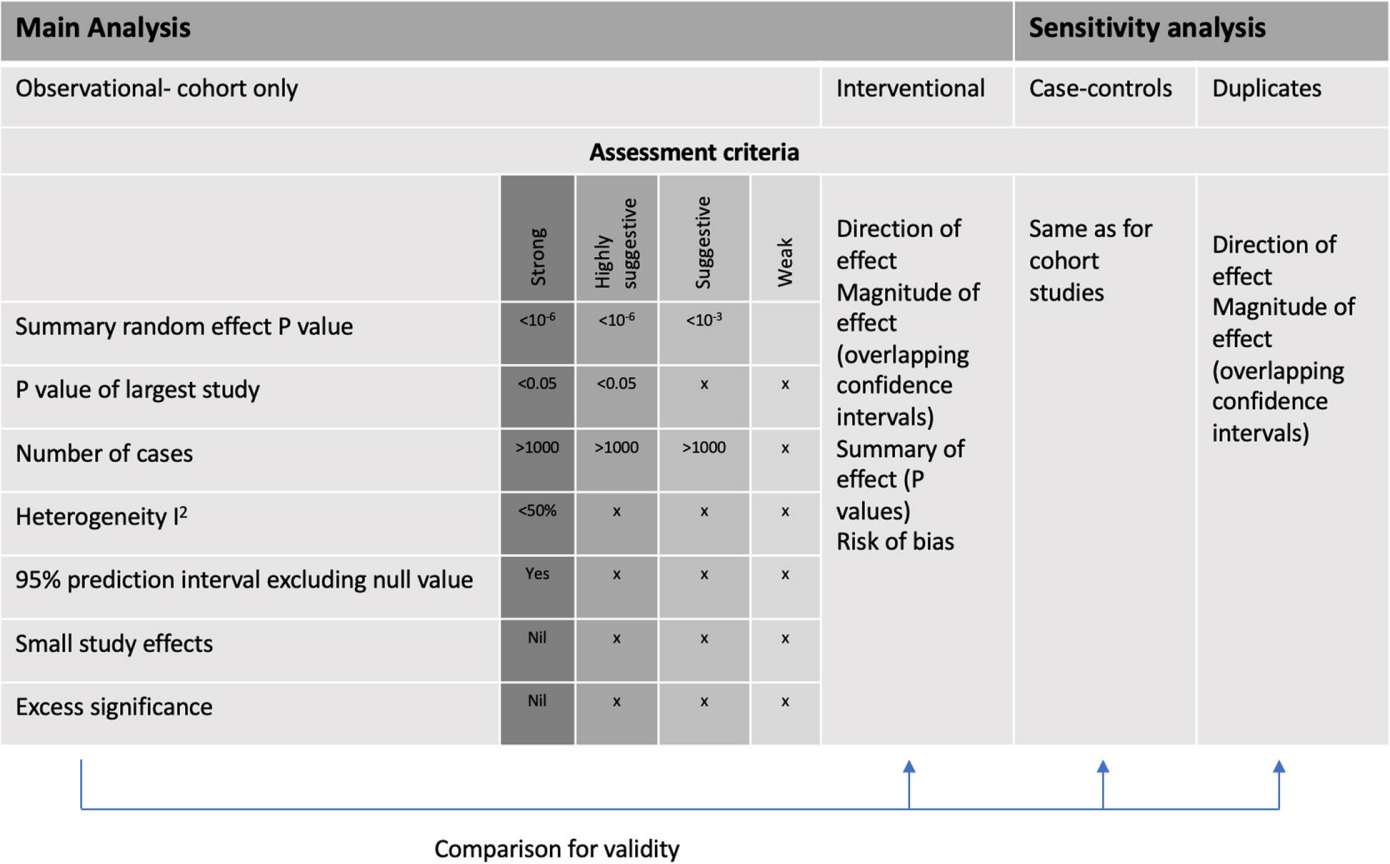
Graphical presentation of main and sensitivity analyses of meta-analyses investigating diabetes and anti-diabetic interventions and gynaecological/obstetric conditions

**Fig. 2:**
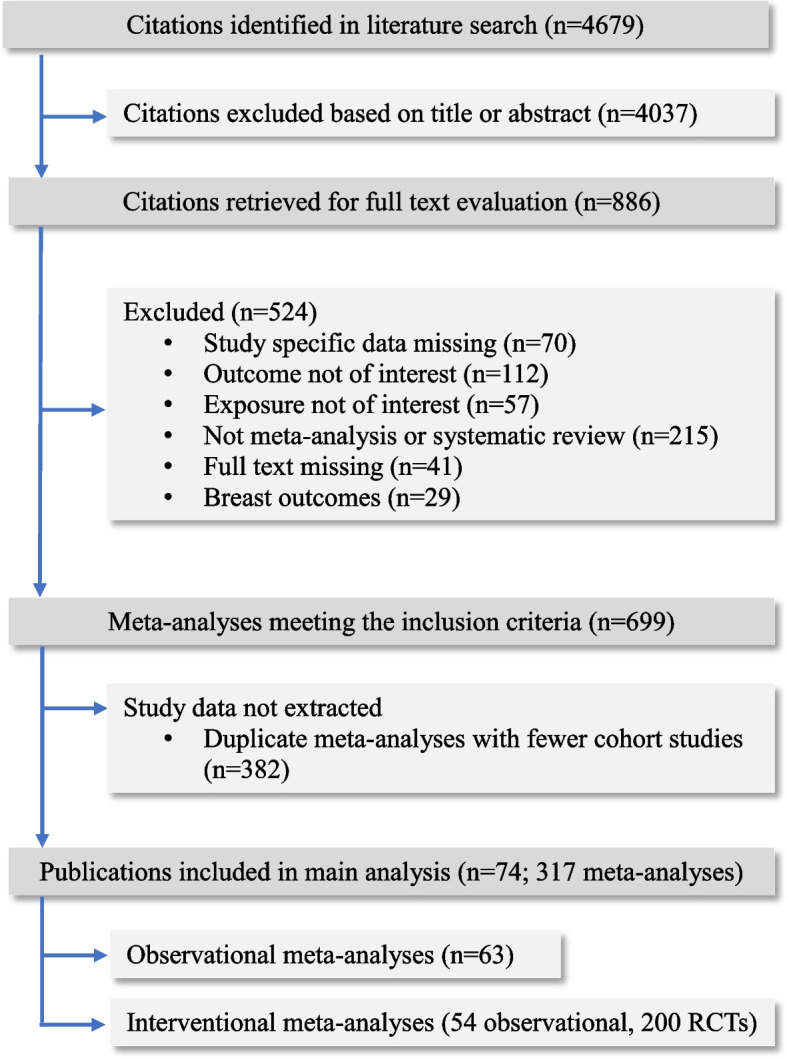
Flow diagram of the selection process of meta-analyses included in the review

A literature search was performed on PubMed, MEDLINE, Embase and Cochrane Database of Systematic Reviews from inception to March 2020 for systematic reviews and meta-analyses published in English on the association of diabetes with gynaecological and obstetric morbidity, i.e. adverse outcomes related to conditions of the female genital tract and pregnancy. Diabetes encompassed diabetes type 1, type 2 and gestational diabetes. The search algorithm did not include diabetes as part of metabolic syndrome. We further searched for meta-analyses of randomised or non-randomised controlled trials that investigated the impact of anti-diabetic regimens on gynaecological and obstetric outcomes. All primary and secondary outcomes related to gynaecological and obstetric morbidity were extracted from original meta-analyses. The search algorithm can be found in Additional material (Additional file 1). Data extraction and analysis strategies can be found in Figs. 1 and 2.

### Inclusion and exclusion criteria

We included systematic reviews and meta-analyses of observational studies and randomised/non-randomised controlled trials conducted in humans that investigated the association of diabetes and anti-diabetic interventions with several gynaecological and obstetric outcomes. We excluded meta-analyses where exposure was hyperglycaemia, impaired glucose tolerance or glycaemic index/load and outcomes that were not pertinent to obstetrics and gynaecology (e.g. non-gynaecological cancer including breast cancer, childhood outcomes in children born to diabetic mothers). We additionally excluded meta-analyses that lacked a comparison arm, meta-analyses assessing diabetes as an outcome and meta-analyses with study-specific data (number of incident events, number of study population or person years, relative risks and 95% confidence intervals) missing from original papers and which could not be retrieved after contacting authors. In cases where more than one meta-analysis existed for the same exposure-outcome association, the meta-analysis with the largest number of cohort studies was selected. In a sensitivity analysis, we further assessed whether there were any differences in the summary findings of duplicate meta-analyses.

### Evaluation of the strength of evidence in observational studies

The robustness of evidence of the reported associations in observational studies was assessed based on previously described criteria [19, 20], which form four grades of descending strength: strong, highly suggestive, suggestive and weak (Fig. 1).

### Evaluation of the methodological quality of included meta-analyses

To assess the methodological quality of observational meta-analyses, we used the AMSTAR (A MeaSurement Tool to Assess Systematic Reviews) 2 tool, which uses 16 measures to classify systematic reviews into high, moderate, low or critically low quality [21]. To assess the methodological quality of interventional meta-analyses of randomised controlled trials, we extracted and presented the risk of bias tool used in each original meta-analysis, where available.

### Data analysis

Our primary analysis focused on meta-analyses of cohort studies that represent the most reliable evidence among observational studies. We additionally performed sensitivity analyses that included both cohort and case-control studies. Furthermore, we applied the credibility ceilings threshold to account that any single observational study cannot give more than a maximum certainty, c% (called credibility ceiling) that the true effect size is in a different direction from the one suggested by the point estimate [22].

Evidence from interventional meta-analyses of randomised controlled trials was analysed separately and included only statistically significant studies (random *P* value < 0.05), for which the quality of evidence, as assessed by GRADE, was extracted and presented from original meta-analyses. All statistical analyses were performed using Stata version 15 (College Station, TX) (StataCorp 2015) [23], and all *P* values were two tailed. Figure 1 summarises the applied methods.

## Results

### Meta-analyses of observational studies

#### Characteristics of meta-analyses

We extracted data from 21 eligible publications, consisting of 63 meta-analyses with 478 study estimates, which investigated the effect of diabetes on gynaecological and obstetric morbidity (Fig. 2). Out of the 478 individual studies, 345 (72%) were cohort studies, 66 (14%) case-control studies, 2 (0.4%) were nested case-control studies, 61 (13%) cross-sectional, 2 (0.4%) descriptive cohort studies and 2 (0.4%) case series. There were between 2 to 31 individual studies per meta-analysis with a median of 13. The median number of cases and total population in each meta-analysis was 1301 and 33,788, respectively. The lowest number of cases in a meta-analysis was 58 and the highest was 251,558, whereas the smallest total population or controls was 272 and the highest was 15,009,001.

#### Summary effect size

Using *P* < 0.05 as a statistical significance threshold, the summary fixed effects estimate reached significance in 35/47 (74%) meta-analyses of cohort studies, while the summary random effects was significant in 33/47 (70%). At a significance cut-off of *P* < 0.001, 27/47 (57%) and 19/47 (40%) meta-analyses yielded significant results using the fixed and random effects model respectively, whereas at a more stringent threshold of *P* < 10^−6^, 22/47 (47%) and 7/47 (15%) of meta-analyses produced significant results. The 7 meta-analyses with strongly statistically significant summary random effect estimates (*P* < 10^−6^) identified a relationship between GDM and increased risk for caesarean section, large for gestational age (LGA) babies, macrosomia and brachial plexus palsy as well as a relationship between pregestational diabetes and increased risk for major congenital malformations, and congenital heart defects (Additional file 2: Table S1A) [3–18, 24–28].

In total, 49% (23/47) of the summary random effect estimates fell between 1.2 and 2. The largest summary random effect (4.53, 1.61 to 12.77) was noted for the risk of major congenital malformations in women with diabetes type 1 or 2 (poor vs optimal glycaemic control). Table S1A reports the effect of the largest study included in each meta-analysis. Fifty-five percent (26/47) of these effects were nominally significant at *P* < 0.05 and showed an increased risk.

#### Heterogeneity between studies

We used the Cochran’s *Q* test and *I*^*2*^ index as measures of between-study heterogeneity to account for variation in study outcomes between studies that cannot be attributed to chance. The *Q* test was significant (*P* ≤ 0.10) for 40% (19/47) of meta-analyses. Moderate to high heterogeneity (*I*^2^ = 50-75%) was noted in 8/47 (17%) of meta-analyses, while substantial heterogeneity (*I*^2^ > 75%) was observed in 10/47 (21%). We further calculated 95% prediction intervals and found 10 associations in which the null value was excluded (Additional file 3: Table S1B) [3–18, 24–28].

#### Small study effects

Small study effects (Egger’s test *P* value < 0.10 and where more conservative effects in the largest study of a meta-analysis compared to the summary random effects estimate were recorded) were found to be present in five meta-analyses for associations between diabetes and postnatal depressive symptoms (GDM vs non-GDM), congenital malformations (poor vs optimal glycaemic control), brachial plexus palsy (GDM vs non-GDM), cervical cancer screening (DM vs non-DM) and neonatal mortality (DM2 vs DM1, not significant) (Additional file 3: Table S1B). Only two studies included an adequate number of studies (10 or more) for Egger’s test to have adequate statistical power to identify small study effects.

#### Excess significance bias

Two (2/47, 4%) meta-analyses demonstrated evidence of excess significance bias using the largest study estimate as the plausible effect size (*P* < 0.10). These included the associations between gestational diabetes and postnatal depressive symptoms and pre- or gestational diabetes with respiratory distress syndrome of the newborn. Using the random effects estimate, the association of GDM with breastmilk energy was additionally highlighted with excess significance bias, while using the summary fixed effects estimate no further meta-analysis was highlighted (Additional file 3: Table S1B).

#### Quality assessment

The methodological quality of the 21 publications included in main analysis, encompassing 47 meta-analyses of observational studies, was assessed using the AMSTAR 2 tool. Three out of the 21 included papers were graded as low and eighteen as critically low quality (Additional file 4: Table S2) [3–18, 24–44]. Papers assessed with ‘low’ or ‘critically low’ quality failed to meet one or more than one ‘critical’ criteria respectively, for example lack of protocol, description of excluded studies or risk of bias assessment. Most publications (19/21) did not meet the critical requirement of explicitly stating that the review methods were established prior to the conduct of the review and failed to justify any significant deviations from the protocol, while 18/21 did not provide a list of excluded studies accompanied by justification. With regard to other critical criteria, all included papers provided a comprehensive literature search strategy and used appropriate statistical analyses for combination of results. 10/21 assessed the risk of bias, whereas 17/21 accounted for publication bias (small study bias).

#### Grading of evidence

Each of the outcomes identified as being associated with diabetes was graded into four groups according to the strength of reported evidence in cohort studies: strong, highly suggestive, suggestive or weak evidence (Table 1). Detailed explanation of the assessment criteria is presented in Additional file 5: Table S3 (for cohort studies only) [3–18, 24–28], while the results for both cohort and case–control studies are shown in Additional file 6: Table S4 [3–18, 24–28, 45, 46].

**Table 1:** Summary of evidence grading for meta-analyses associating diabetes and anti-diabetic interventions with risk of obstetric and gynaecological morbidity—cohort studies only

**Evidence**	**Criteria used**	**Decreased risk**	**Increased risk**
**Strong**	*P* < 10^−6**||**^; > 1000 cases; *I*^2^ < 50%; no small study effects^¶^; prediction interval excludes the null value; no excess significance bias^†^ survives 10% credibility ceiling *n* = 2		**Maternal outcomes** ⦁ Caesarean section (GDM vs non-GDM)
			**Fetal outcomes** ⦁ Large for gestational age (GDM vs non-GDM)
**Highly suggestive**	*P* < 10^−6**||**^; > 1000 cases; *P* < 0.05 of the largest study in a meta-analysis *n* = 3	**Gynaecological outcomes** ⦁ Ovarian cancer occurrence (Metformin vs non-metformin, DM2)	**Fetal outcomes** ⦁ Major congenital malformations (unspecified) (PGDM vs non-DM) ⦁ Congenital heart defects (PGDM vs non-DM)
**Suggestive**	*P* < 10^−3**||**^; > 1000 cases *n* = 10		**Maternal outcomes** ⦁ Preeclampsia (GDM vs non-GDM, IADPSG criteria) ⦁ Preeclampsia (GDM vs non-GDM, WHO criteria) ⦁ Postnatal depression (GDM vs non-GDM)
			**Fetal outcomes** ⦁ Stillbirth (> 20 weeks or > 400 g) (PGDM vs non-DM) ⦁ Major congenital malformations (unspecified) (GDM vs non-GDM) ⦁ Large for gestational age (GDM vs non-GDM, IADPSG criteria) ⦁ Respiratory distress syndrome (DM vs non-DM) ⦁ Introduction of formula milk/breastmilk substitute before hospital discharge (GDM vs non-GDM)
			**Gynaecological outcomes** ⦁ Endometrial cancer incidence (DM vs non-DM) ⦁ Improved endometrial cancer survival (metformin vs other anti-diabetics)
**Weak**	*P* < 0.05 *n* = 28		**Maternal outcomes** ⦁ Miscarriage (poor vs optimal glycaemic control of DM1/2) ⦁ Antenatal depressive symptoms ⦁ (GDM vs non-GDM) ⦁ Decreased duration of ⦁ breastfeeding (GDM vs non-GDM) ⦁ Low breastmilk protein content (GDM vs non-GDM) ⦁ Miscarriage (continuous sc Ins infusion vs multiple daily inj, DM1)
		**Fetal outcomes** ⦁ Congenital malformations (preconception vs no preconception care, PGDM) ⦁ Perinatal mortality (preconception vs no preconception care, PGDM) ⦁ Preterm delivery (preconception vs no preconception care, PGDM)	**Fetal outcomes** ⦁ Macrosomia (GDM vs non-GDM, WHO criteria) ⦁ Macrosomia in Iranian women (GDM vs non-GDM) ⦁ Congenital malformations (unspecified) (poor vs optimal glycaemic control of DM1/2) ⦁ Major congenital malformations (poor vs optimal glycaemic control of DM1/2) ⦁ Perinatal mortality (poor vs optimal glycaemic control of DM1/2) ⦁ Perinatal mortality (DM2 vs DM1) ⦁ Brachial plexus palsy (GDM vs non-GDM) ⦁ Respiratory distress syndrome (GDM vs non-GDM) ⦁ LGA (Lispro vs Regular Ins or NPH, GDM, DM1/2) ⦁ LGA (Lispro vs Regular Ins, DM1) ⦁ Macrosomia > 4.5 kg (continuous sc Ins infusion vs multiple daily inj, DM1) ⦁ Higher birth weight (Lispro vs Regular Ins or NPH, GDM/ DM1/2)
		**Gynaecological outcomes** ⦁ Cervical cancer occurrence (metformin vs non-metformin, T2DM)	**Gynaecological outcomes** ⦁ Endometrial cancer mortality (disease-specific) (DM1/2 vs non-DM) ⦁ Premalignant/malignant endometrial polyps (DM vs non-DM) ⦁ Ovarian cancer incidence (DM vs non-DM) ⦁ Ovarian cancer mortality (disease-specific) (DM1/2 vs non-DM) ⦁ Infrequent cervical cancer screening (DM vs non-DM) ⦁ Ovarian cancer incidence (DM1 vs non-DM) ⦁ Ovarian cancer incidence (DM2 vs non-DM)

*Abbreviations*: *GDM*, gestational diabetes mellitus; *PGDM*, pregestational diabetes mellitus; *DM1/2*, type 1/2 diabetes mellitus; *LGA*, large for gestational age; *CS*, caesarean section; *WHO*, World Health Organization; *IADSPG*, International Association of Diabetes and Pregnancy Groups; *Ins*, insulin; *sc*, subcutaneous; *inj*, injections

Only meta-analyses meeting at least weak grade of evidence listed

^**||**^*P* indicates the *P*-values of the meta-analysis random effects model

^¶^Small study effect is based on the *P*-value from the Egger’s regression asymmetry test (*P* > 0.1) where the random effects summary estimate was larger compared to the point estimate of the largest study in a meta-analysis

^†^Based on the *P*-value (*P* > 0.1) of the excess significance test using the largest study (smallest standard error) in a meta-analysis as the plausible effect size

Only two out of 47 (4%) meta-analyses fulfilled the criteria of strong evidence of an association with diabetes; these reported an association between GDM and increased risk of caesarean section (GDM vs non-GDM, RR 1.37, 95% CI 1.24 to 1.51) and LGA (GDM vs non-GDM, RR 1.53, 95% CI 1.39 to 1.69). Highly suggestive evidence was presented by two meta-analyses (4%), which reported associations between pre-gestational diabetes and increased risk of major congenital malformations (PGDM vs non-DM, RR 2.44, 95% CI 1.92 to 3.10) and congenital heart defects (PGDM vs non-DM, OR 3.18, 95% CI 2.77 to 3.65). Nine meta-analyses (19%) described suggestive evidence for associations between diabetes and several outcomes, including pre-eclampsia, stillbirth (> 20 weeks or > 400 g), postnatal depression, respiratory distress syndrome, introduction of breastmilk substitute before hospital discharge and endometrial cancer. An additional 20 meta-analyses (43%) described weak evidence, whereas 14 meta-analyses (30%) showed no association (*P* > 0.05).

#### Sensitivity analysis

When both cohort and case-control studies were included in the analysis, two further meta-analyses met the criteria for strong evidence; these reported the association of PGDM and stillbirth (> 20 weeks or > 400 g), which was upgraded from suggestive and the association of DM and infrequent cervical cancer screening, previously classified as weak evidence. The association of GDM and brachial plexus palsy was upgraded from weak to highly suggestive, while the association of GDM with respiratory distress syndrome was upgraded from weak to suggestive (Additional file 6: Table S4).

We identified 24 duplicate meta-analyses investigating the same exposure and outcome association that were not selected for further analysis either because of missing study specific data (number of cases/controls, relative risk, 95% confidence interval) or because another study included a larger number of cohort studies (Additional file 7: Table S5) [3–11, 14, 15, 17, 18, 26, 27, 47–57]. For the majority of these duplicate meta-analyses, there was agreement in principle on the direction, magnitude, and significance of the summary associations with the meta-analyses included in the analysis instead.

#### Credibility ceilings

From all 47 meta-analyses, 29 (62%) met nominal significance (*P* < 0.05) with a credibility ceiling of 5%. With ceilings of 10%, 15%, and 20%, 24 (51%), 9 (19%), and 4 (8.5%) meta-analyses remained significant, respectively (Additional file 3: Table S1B).

### Meta-analyses of interventional studies

We identified 254 meta-analyses of interventional studies from 49 publications, including 54 observational studies (49 cohorts) and 200 randomised controlled trials, which examined the effect of anti-diabetic interventions on gynaecological and obstetric conditions (Fig. 2).

#### Observational interventional

##### Characteristics of meta-analyses

We extracted data from 16 eligible publications, consisting of 54 meta-analyses with 266 study estimates, which investigated the effect of anti-diabetic interventions on gynaecological and obstetric morbidity. Out of the 266 individual studies, 200 (75%) were cohort studies, 44 (17%) case-control studies, while the rest represented other study types. There were two to 16 individual studies combined per meta-analysis with a median of 6. The median number of cases and total population in each meta-analysis was 121 and 638 respectively. The lowest number of cases in a meta-analysis was 8 and the highest was 8723, whereas the smallest total population or controls was 103 and the highest was 5,295,969.

##### Summary effect size

Using *P* < 0.05 as a statistical significance threshold, the summary fixed effects estimate reached significance in 17/49 (35%) meta-analyses of cohort studies, while the summary random effects was significant in 11/49 (22%). At a significance cut-off of *P* < 0.001, 9/49 (18%) and 5/49 (10%) meta-analyses yielded significant results using the fixed and random effects model respectively, whereas at a more stringent threshold of *P* < 10^−6^, 4/49 (8%) and 2/49 (4%) of meta-analyses produced significant results. The two meta-analyses with strongly statistically significant summary random effect estimates (*P* < 10^−6^) reported a decreased risk of ovarian cancer occurrence in metformin users of DM2 versus non-metformin users and a decreased risk of congenital malformations in women with PGDM who received preconception care (Additional file 8: Table S6A) [29–42, 44]. The summary random effect estimates ranged from 0.18 (95% confidence interval 0.12 to 0.25) for an association between metformin use and ovarian cancer occurrence up to 92.22 (− 73.15 to 257.59) for an association between insulin analogue use and birth weight.

##### Heterogeneity between studies

The *Q* test was significant (*P* ≤ 0.10) for 16% (8/49) of meta-analyses. Moderate to high heterogeneity (*I*^2^ = 50–75%) was noted in 4/49 (8%) of meta-analyses, while substantial heterogeneity (*I*^2^ >75%) was observed in 3/49 (6%) (Additional file 9: Table S6B) [29–42, 44].

##### Small study effects

Small study effects (Egger’s test *p* value < 0.10 and where more conservative effects in the largest study of a meta-analysis compared to the summary random effects estimate were recorded) was found to be present in one meta-analysis, consisting of only 3 individual studies and describing a reduced risk of preterm delivery in type 1 diabetics treated with multiple daily insulin injections versus continuous subcutaneous infusion (Additional file 9: Table S6B).

##### Excess significance bias

One meta-analysis demonstrated evidence of excess significance bias using the largest study estimate as the plausible effect size (*P* < 0.10), which reported deceased risk of congenital malformations in women receiving preconception care and decreased risk of preeclampsia in DM1 patients using continuous subcutaneous insulin infusion (vs multiple daily injections). Using the random effects estimate, the association of metformin use with increased endometrial cancer occurrence and survival were also highlighted with excess significance bias, while using the summary fixed effects estimate no further meta-analysis was highlighted (Additional file 9: Table S6B).

##### Quality assessment

The methodological quality of the 16 publications included in main analysis, encompassing 49 meta-analyses of interventional observational studies, was assessed using the AMSTAR 2 tool. One (1/16) of all the included papers was graded as low and fifteen (15/16) as critically low quality (Additional file 4: Table S2). Most publications (12/16) did not meet the critical requirement of explicitly stating that the review methods were established prior to the conduct of the review and failed to justify any significant deviations from the protocol, while 13/16 did not provide a list of excluded studies accompanied by justification. With regard to other critical criteria, most included papers (12/16) provided a comprehensive literature search strategy and used appropriate statistical analyses for combination of results (12/14). 5/16 assessed the risk of bias, whereas 8/16 accounted for publication bias (small study bias).

##### Grading of evidence

With regard to anti-diabetic interventions, no association was supported by strong evidence, while a single (1/49) meta-analysis presented highly suggestive evidence reporting a decreased risk of ovarian cancer in metformin users of DM2 versus non-metformin users (OR 0.55, 95% CI 0.36 to 0.84). Suggestive evidence was presented by one meta-analysis demonstrating improved endometrial cancer survival in diabetic patients using metformin versus other anti-diabetics (HR 0.47, 95% CI 0.33 to 0.67). Nine meta-analyses (18%) described weak evidence for several outcomes, including metformin use and decreased cervical cancer occurrence, preconception care in PGDM and decreased rate of congenital malformations, perinatal mortality and preterm delivery (Table 1). Thirty-eight (38/49, 78%) meta-analyses showed no association (*P* > 0.05) between anti-diabetic interventions and gynaecological/obstetric outcomes.

##### Sensitivity analysis

When both cohort and case-control studies were included in the analysis, the association of myoinositol use with reduced birth weight in GDM was upgraded from non-significant to weak.

##### Credibility ceilings

From all 49 meta-analyses, 9 (18%) met nominal significance (*P* < 0.05) with a credibility ceiling of 5%. With a ceiling of 10%, 3 (6%) meta-analyses remained significant, whereas no meta-analysis survived the 15% and 20% credibility ceilings (Additional file 9: Table S6B).

##### Randomised controlled trials (RCTs)

We retrieved 200 meta-analyses of randomised controlled trials from 38 publications describing the effect of lifestyle interventions (*n* = 20), dietary interventions (*n* = 25), exercise (*n* = 4), oral anti-diabetics and/or insulin (*n* = 110) and omega-3 supplements (*n* = 4) on 37 outcomes.

We observed nominally significant associations (*P* random value < 0.05) in 40/200 (20%) meta-analyses for 16 associations on 16 separate outcomes: between lifestyle interventions and reduced rates of LGA, macrosomia and shoulder dystocia; between metformin (vs insulin) and reduced rates of LGA and macrosomic babies, caesarean section, gestational hypertension, pregnancy-induced hypertension, preeclampsia, neonatal intensive care unit (NICU) admission, neonatal hypoglycaemia and hyperbilirubinaemia; between any specific/intensive treatment and reduction of neonatal hypoglycaemia, preeclampsia, shoulder dystocia, hypertensive disorders of pregnancy, LGA, macrosomia and increased rates of induction of labour (Additional file 10: Table S7) [58–75].

Interventional meta-analyses (RCTs) reached statistical significance for both outcomes that met the strong criteria in observational studies. Metformin and internet-based self-monitoring were found to reduce the risk of caesarean section among women with GDM versus insulin and usual care respectively. Decreased rates of LGA were also demonstrated when metformin or GDM treatment were used versus insulin and usual antenatal care respectively. No RCTs have been published for highly suggestive outcomes (major congenital malformations and heart defects in pre-gestational diabetes), but one observational interventional study showed weak evidence of preconception care reducing the risk of congenital abnormalities in patients with PGDM. With regard to suggestive outcomes, metformin or any treatment were nominally significant in reducing the risk of preeclampsia when compared to insulin and no treatment respectively. No RCTs were retrieved on diabetes and gynaecological outcomes.

Altogether, 16 out of 17 publications that yielded statistically significant results assessed the risk of bias of the included studies (Additional file 11: Table S8) [58–66, 68–74]. All publications used the Cochrane risk of bias tool or a modification of this (Risk of bias assessment tool recommended by the Cochrane Neonatal Review group) [76–78]. The GRADE score [79] was used to assess the quality of evidence in five meta-analyses. Five publications used more than one tool to assess methodological quality, and one publication used none. For outcomes that were assessed by GRADE method, high-quality evidence supported the association between GDM treatment and reduced rate of LGA/macrosomia; moderate-quality evidence demonstrated a reduction in LGA when lifestyle interventions were in place and a drop in rates of hypertensive disorders of pregnancy when GDM was treated; finally, low- and very low-quality of evidence supported the link between GDM treatment and reduced shoulder dystocia, metformin and reduced caesarean sections and hypertensive disorders of pregnancy, dietary interventions (DASH, soy protein) and reduced birth weight (Additional file 10: Table S7).

## Discussion

This umbrella review represents the most comprehensive overview of published literature to date investigating associations between diabetes or anti-diabetic interventions and the risk of any type of obstetric or gynaecological morbidity. We included 117 meta-analyses of observational studies and 200 meta-analyses of clinical trials that evaluated 317 outcomes, which were subsequently graded based on the strength of association (observational only). Strong evidence of association was observed for only two outcomes; women with GDM seem to be at higher risk of caesarean section and LGA. LGA is explained pathophysiologically by the Pedersen hypothesis according to which maternal hyperglycaemia leads to fetal hyperglycaemia and hyperinsulinaemia, which incites fetal overgrowth [80]. When case-control studies were included in the analysis, two further associations were considered strong; PGDM and stillbirth (> 20 weeks or > 400 g) as well as DM and infrequent cervical screening. The positive association of PGDM with major congenital malformations and congenital heart defects was graded as highly suggestive, while the evidence linking GDM with brachial plexus palsy was also upgraded to highly suggestive, when case control studies were included. Animal studies have showed that PGDM induces cellular oxidative stress, impairs endogenous antioxidant capacity and triggers apoptotic pathways in target organs resulting in structural birth defects, including the embryonic heart and neural tube [81].

Eight obstetric outcomes were supported by suggestive evidence including preeclampsia, postnatal depression, and neonatal respiratory distress syndrome. Increased oxidative stress, placental endothelial dysfunction and dysregulated angiogenesis underpin both preeclampsia and GDM suggesting a potential link [82]. Surprisingly, the link of macrosomia with GDM was only supported by weak evidence. This finding partly contradicts a Mendelian randomisation study which revealed genetic evidence for a possible causal association between higher maternal fasting glucose and higher birth weight and also the HAPO study that demonstrated a higher risk of birth weight above the 90th centile (OD 1.38, 95% CI 1.32 to 1.44) in women with fasting hyperglycaemia [83, 84]. The discrepancy noted could be potentially attributed to the fact that our analysis included diabetes as exposure and not hyperglycaemia, which may be left untreated.

Suggestive evidence supported increased endometrial cancer incidence among diabetic patients and increased endometrial cancer survival in metformin users, while the evidence showing an association of diabetes with endometrial cancer mortality was weak. The results are consistent with the 2018 report of the World Cancer Research Fund Continuous Update Project (WCRF CUP), which identifies glycaemic load as a probable cause of endometrial cancer [85]. Interestingly, a large prospective cohort study using the Women’s Health Initiative (WHI) dataset and encompassing 88,107 postmenopausal participants, concluded that the significant higher risk of endometrial cancer in diabetic patients (HR = 1.44, 95% CI: 1.13 to 1.85 for diabetes, HR = 1.57, 95% CI: 1.19 to 2.07 for treated diabetes) was rendered non-significant when adjusting for BMI [86]. This finding suggests that confounders should be considered before establishing associations. In addition to this, a Mendelian randomisation study did not demonstrate an association between DM2 variants and endometrial cancer but supported a causal association of hyperinsulinaemia with endometrial cancer risk, irrespectively of BMI [87].

The association of ovarian cancer incidence and mortality with diabetes was supported by weak evidence irrespective of exposure (DM1, DM2 or both). However, ever use of metformin demonstrated highly suggestive evidence for a lower ovarian cancer risk (RR 0.18, 95% CI 0.12 to 0.25). A proposed mechanism of the antineoplastic effect of metformin is by diminishing cell growth and proliferation through the regulation of the mitogenic IGF1/AKT/mTOR pathway [88]. On the other hand, other epidemiological studies have concluded that metformin does not affect cancer risk for any type of cancer [89]. Caution should be exercised, however, when analysing evidence from observational studies of intervention considering that by definition treatment allocation cannot be random and is more likely to be dictated by patient characteristics, thus introducing biases.

Forty out of 200 meta-analyses of randomised controlled trials (20%) exploring the impact of anti-diabetic interventions on gynaecological and obstetric adverse outcomes reached nominal significance for 18 outcomes. GDM treatment is associated with a reduced risk of preeclampsia, macrosomia, LGA, shoulder dystocia, neonatal hypoglycaemia; insulin is associated with decreased rates of macrosomia versus diet and increased neonatal intensive care unit admission versus metformin; metformin is linked with reduced rates of hypertensive disorders in pregnancy, caesarean section, hyperbilirubinaemia, LGA, neonatal hypoglycaemia and NICU admission compared to insulin; lifestyle interventions correlate with reduced birth weight, LGA, macrosomia and shoulder dystocia, while dietary interventions (DASH diet, soy protein) with reduced birth weight.

### Strengths and limitations

This umbrella review represents the most comprehensive overview of published literature to date investigating associations between diabetes or anti-diabetic interventions and the risk of any type of obstetric or gynaecological morbidity. The strength and validity of meta-analyses of observational studies was assessed against a transparent and replicable set of statistical criteria that categorised observational evidence as strong, highly suggestive, suggestive and weak, while randomised controlled trials were evaluated based on the statistical significance of associations and reporting of risk of bias and quality of evidence by the original meta-analyses.

Our review has a few inherent limitations that should be considered when interpreting the findings. Our analysis relied on previously published meta-analyses and literature searches performed by the authors of meta-analyses; therefore, some individual studies might have been missed. Nevertheless, this is unlikely to have significantly influenced our findings because the assessment of duplicate meta-analyses on the same associations between exposure-outcome pairs reported similar summary results. In addition to this, reporting bias by the authors of meta-analyses could have led to underreporting of associations that did not reach statistical significance or did not conform with general knowledge and expectations [90, 91]. Furthermore, some associations were derived from a small number of studies included in a meta-analysis making the assessment of small study effects and excess significance bias potentially misleading given the low statistical power. Considering the large observed heterogeneity and some hints of bias in several of these meta-analyses, false-positives and inflated results could not be definitively excluded.

Even though a wide range of statistical tools was used to explore risk of bias in individual primary studies of a meta-analysis, other types of biases may have been overlooked and not detected by the statistical tests used. For example, confounding bias may have undermined the reliability of associations [92, 93]. Absence of patient stratification and statistical adjustment for imbalances in risk factors such as obesity, commonly encountered in diabetic patients, or age, diet, physical activity, alcohol, smoking, co-morbidities and drug use could have impacted on observed associations and shifted relationships. Finally, lack of clear definition or consistency of the clinical criteria used for diagnosing diabetes and several outcomes, e.g. macrosomia, stillbirth, congenital malformations, may have led to spurious data collation. In the same context, ambiguity remains on whether epidemiological studies were based on self-report questionnaires or medical records of diabetes given that a study has showed that self-report of diabetes is < 66% sensitive compared to medical record data [94].

## Conclusions

This umbrella review provides a comprehensive summary of the published body of evidence on gynaecological and obstetric ramifications of the diabetes epidemic. There is a strong association between GDM and increased risk of caesarean section and LGA, while interventional studies demonstrated that metformin mitigates the risk for both outcomes. Moreover, metformin displayed superiority to insulin in reducing the risk of hypertensive disorders in pregnancy, hyperbilirubinaemia, neonatal hypoglycaemia and NICU admission. Highly suggestive evidence supports the association of PDGM and increased risk of major congenital malformations and heart defects. Weaker associations were demonstrated for remaining outcomes that could still be valid but mandate further investigation.

The identification of robust relationships between diabetes and gynaecological and obstetric outcomes, following stringent scrutiny of studies against clearly defined statistical criteria that account for small sample size and potential bias, is crucial to reveal actionable risk factors and best interventional strategies. This umbrella review may help guide targeted prevention initiatives, further epidemiological studies with standardised design and reporting of analysis but also future studies designed to investigate the underlying molecular mechanisms of diabetes-induced adverse outcomes.

## Research in context summary

- Diabetes represents a global public health issue and has been associated with a number of adverse obstetric outcomes and gynaecological conditions.
- Several randomised controlled trials have investigated the effect of anti-diabetic lifestyle, dietary or drug interventions on a number of these outcomes.
- Although some of the proposed associations may be causal, methodological pitfalls, inherent bias and differences in the quality of evidence of observational and interventional meta-analyses might obscure true associations.
- This umbrella review includes 117 meta-analyses of observational cohort studies and 200 meta-analyses of randomised clinical trials that evaluated 317 outcomes and provides the most comprehensive appraisal of the literature to date exploring the strength and validity of the published associations between diabetes and anti-diabetic interventions on obstetric and gynaecological morbidity.
- We found strong evidence to support the association between gestational diabetes with risk of caesarean section and large for gestational age babies, while supportive evidence linked diabetes with endometrial cancer incidence.
- High-quality evidence supported an association between gestational diabetes treatment and reduced rates of large for gestational age babies and macrosomia.
- Metformin use in gestational or pregestational diabetes is linked with reduced rates of hypertensive disorders in pregnancy, caesarean section, hyperbilirubinaemia, large for gestational age babies, neonatal hypoglycaemia and neonatal intensive care unit admission compared to insulin.
- This study will inform clinical practice by highlighting the obstetric and gynaecological outcomes that are robustly linked to diabetes and the type of interventions that have proven efficacy in preventing its deleterious effects.

## Supplementary Information

**Additional file 1.** Search Algorithm.

**Additional file 2: Table S1A.** Description of 47 meta-analyses results investigating the association of diabetes with gynaecological and obstetric morbidity- cohort studies only.

**Additional file 3: Table S1B.** Evaluation of heterogeneity, small study effects, excess significance bias and credibility ceilings in the 47 meta-analyses investigating the association of diabetes with gynaecological and obstetric morbidity– cohort studies only.

**Additional file 4: Table S2.** AMSTAR 2 methodological quality assessment for observational systematic reviews investigating the association of diabetes and anti-diabetic interventions with gynaecological and obstetric morbidity- cohorts only.

**Additional file 5: Table S3.** Details of evidence grading for meta-analyses associating diabetes and anti-diabetic interventions with risk of obstetric and gynaecological morbidity– cohort studies only.

**Additional file 6: Table S4.** Details of evidence grading for meta-analyses associating diabetes and anti-diabetic interventions with risk of obstetric and gynaecological morbidity– all study types included.

**Additional file 7: Table S5.** Duplicate and excluded meta-analyses and meta-analyses included instead on the effect of diabetes on gynaecological/obstetric outcomes- cohorts only.

**Additional file 8: Table S6A.** Description of 49 meta- analyses results investigating the association of anti-diabetic interventions with gynaecological and obstetric morbidity–cohort studies only.

**Additional file 9: Table S6B.** Evaluation of heterogeneity, small study effects, excess significance bias and credibility ceilings in the 49 meta-analyses investigating the association of anti-diabetic interventions with gynaecological and obstetric morbidity– cohort studies only.

**Additional file 10: Table S7.** Meta-analyses including only statistically significant* RCTs over the effect of any anti-diabetic intervention on the incidence of any obstetric or gynaecological disease.

**Additional file 11: Table S8.** Risk of bias in the included interventional meta-analyses (RCTs).

## Data Availability

All data were collected from publicly available literatures and all data generated or analysed during this study are included in this published article and its additional files. This study was conducted in accordance with the PRISMA guidelines [95].

## Abbreviations

DM: Diabetes mellitus
DM1: Diabetes mellitus type 1
DM2: Diabetes mellitus type 2
PGDM: Pregestational diabetes mellitus
GDM: Gestational diabetes mellitus
AMSTAR: A MeaSurement Tool to Assess Systematic Reviews
LGA: Large for gestational age
NICU: Neonatal intensive care unit
RCTs: Randomised controlled trials
