# Supplementary material for: Diabetes and anti-diabetic interventions and the risk of gynaecological and obstetric morbidity: an umbrella review of the literature
Source: BMC Med. 2023 Apr 18;21:152. doi: 10.1186/s12916-023-02758-1 (PMC10114404; doi:10.1186/s12916-023-02758-1)
Supplement: Supplementary file 1 — Additional file 1. Search Algorithm. [file 12916_2023_2758_MOESM1_ESM.docx]

**Pubmed Search algorithm**

#1 endometrial neoplasm [MeSH]
#2 malign* [tiab] OR cancer*[tiab] OR carcinoma*[tiab] OR tumor*[tiab] OR tumour*[tiab] #3 endometr* [tiab] OR corpus uteri [tiab] OR uterine [tiab]
#4 #2 AND #3
#5 #1 AND # 4

#6 Ovarian Neoplasms [MeSH]
#7 Ovar* AND (cancer* OR carcinoma* OR neoplasm* OR tumor* OR tumour* OR adenocarcinoma* OR Endometrioid carcinoma* OR cystadenoma* OR cystadenocarcinoma* OR adenoma*)
#8 Androblastom* OR arrhenoblastoma* OR sertoli leydig OR Brenner OR granulosa cell tumor* OR granulosa cell tumour* OR luteoma* OR luteinoma*
#9 #6 OR #7 OR #8

#10 Cervical neoplasms [MeSH]
#11 Cervi* AND (cancer* OR carcinoma* OR neoplasm* OR tumor* OR tumour* OR adenocarcinoma* OR squamous cell carcinoma* OR carcinoma OR carcinosarcoma*)
#12 CIN OR cervical intraepithelial neoplasia OR cervical dysplasia OR cervical precancer*
#13 #10 OR #11 OR #12

#14 Pregnancy [mh] OR Pregnant Women [mh] OR pregnan*[tiab] OR Parturition [mh] OR parturi* [tiab] OR gestation* [tiab] OR Gravidity [mh] OR gravid* [tiab] OR maternal* [tiab] OR puerperium [mh] OR puerperi* [tiab] OR postpartum period [mh] OR postpartum* [tiab] OR pregnancy complications [mh] OR pregnancy outcome [mh]

#15 Polycystic ovary syndrome [mh] OR polycystic ovar*[tiab] OR pco [tiab] OR pcos [tiab] OR pcod [tiab]

#16 fertility[mh] OR infertility [mh] OR Abortion, Spontaneous[mh] OR miscarriage [tiab] OR extrauterine pregnancy [tiab] OR ectopic pregnancy [tiab] OR abortion, induced[mh] OR termination of pregnancy[tiab] OR molar pregnancy[tiab] OR IVF[tiab] OR ICSI[tiab] OR insemination[tiab] OR assisted reproduction[tiab] OR pelvic floor disorders[tiab] OR urinary incontinence[tiab] OR fecal incontinence[tiab] OR pelvic organ prolapse[tiab] OR uterine prolapse[tiab] OR vaginal prolapse[tiab] OR pelvic floor defect[tiab] OR pelvic floor*[tiab] OR vaginal wall*[tiab] OR Reproductive Techniques [mh] OR Genital Disease, Female [mh]OR Pelvic Floor Diseases [mh]

#17 menorrhagia[mh] OR metrorragia[mh] OR menstrual cycle[tiab] OR menstrual disorder*[tiab] OR heavy menstrual bleeding[tiab] OR menstrual bleeding[tiab] OR menstrual pain[tiab] OR menstrual cycle*[tiab] OR menopause [mh] OR postmenopaus* [tiab] OR premenopaus* [tiab] OR climacteric [mh]

#18 contracept*[tiab] OR Norpregnanes[mh] OR “Contraceptive Agents” [mh] OR “Contraceptive Agents” [Pharmacological Action] OR “Contraceptive Devices” [mh] OR “Contraception” [mh] OR iud [tiab] OR “Intrauterine Devices” [mh] OR emergency contraception[tiab] OR “Contraception,
Postcoital” [mh] OR “Contraceptive Agents” [mh] OR nuvaring [tiab] OR “Desogestrel”[mh] OR “Contraceptive Agents, Female” [mh] OR “Contraceptive Devices, Female” [mh] OR hormonal patch[tiab] OR ortho evra [tiab] OR “Norgestrel” [mh] OR “Contraceptive Devices, Female” [mh]) OR “Contraceptive Agents, Female” [mh]

#19 Congenital abnormalities [MeSH]
#20 congenital adj3 (abnormalit* OR defect* OR deformit*) OR birth adj3 ( abnormalit* OR defect* OR deformit*)
#21 #19 OR #20

#22: #5 OR #9 OR #13 OR #14 OR #15 OR #16 OR #17 OR #18 OR #21 #23 diabe* [tiab] OR diabetes [MeSH]

#24 #22 AND #23
#25 animal[mh] NOT human[mh]
#26 #24 NOT #25
#27 Meta-Analysis[ptyp] OR systematic[sb] #28 #26 AND #27

**Cochrane database of systematic reviews**

#1"diabetes”
#2 [mh obstetrics] or [mh gynecology]

#3 endometrial or cervical or ovari or birth or maternal or gestati or pregnancy or fertility or menstrual or polycystic or hrt or contracepti
#4 #2 or #3
#5 #4 and #1
