# Supplementary material for: Diabetes and anti-diabetic interventions and the risk of gynaecological and obstetric morbidity: an umbrella review of the literature
Source: BMC Med. 2023 Apr 18;21:152. doi: 10.1186/s12916-023-02758-1 (PMC10114404; doi:10.1186/s12916-023-02758-1)
Supplement: Supplementary file 2 — Additional file 2: Table S1A. Description of 47 meta-analyses results investigating the association of diabetes with gynaecological and obstetric morbidity- cohort studies only. [file 12916_2023_2758_MOESM2_ESM.docx]

**Table S1A: Description of 47 meta-analyses results investigating the association of diabetes with gynaecological and obstetric morbidity–**

**cohort studies only**

| **Author, year** | **Exposure** | **Exposure contrast** | **N**^α^ | **Sample size**  **cases/ cohort** | **Summary relative risk (95% CI)** | | | **Fixed**  **P-value**^ε^ | **Random**  **P-value**^φ^ | **95% Prediction interval**^γ^ |
| --- | --- | --- | --- | --- | --- | --- | --- | --- | --- | --- |
|  |  |  |  |  | **Fixed Effects**^β^ | **Random Effects**^χ^ | **Largest Study**^δ^ |  |  |  |
| **Gynaecological** | | | | | | | | | | |
| **DM** | | | | | | | | | | |
| Saed 2019 | DM | DM vs non- DM | 9 | 3564/429206 | 1.42 (1.25-1.62) | 1.56 (1.21-2.01) | 1.16 (0.90-1.48) | 5.93E-08 | 5.04E-04 | 0.72-3.37 |
| Liao 2014 | DM 1/2 | DM vs non- DM | 6 | 2075/1,268,756 | 1.32 (1.11-1.57) | 1.32 (1.11-1.57) | 1.33 (1.07-1.65) | 1.6E-3 | 1.6E-3 | 1.03-1.69 |
| Pergaliotis 2016 | DM (unspecified) | DM vs non- DM | 8 | 154/5212 | 2.85 (1.72-4.72) | 2.85 (1.72-4.72) | 3.04 (1.02-9.05) | 4.53E-05 | 4.53E-05 | 1.52-5.35 |
| Wang 2017 | DM (unspecified) | DM vs non- DM | 14 | 5534/3708313 | 1.15 (1.07-1.24) | 1.19 (1.06-1.34) | 1.05 (0.93-1.2) | 8.00E-05 | 4.36E-03 | 0.87-1.62 |
| Zhang 2017 | DM 1/2 | DM vs non- DM | 5 | 15312/610592 | 1.31 (1.21-1.42) | 1.44 (1.08-1.93) | 1.32 (1.16-1.5) | 2.09E-10 | 1.35E-02 | 0.49-4.25 |
| Bhatia 2020 | DM | DM vs non- DM | 3 | 2463/4268 | 0.71 (0.52-0.96) | 0.71 (0.52-0.96) | 0.74 (0.50-1.10) | 0.029 | 0.029 | 0.09-5.31 |
| Zhang 2017 | DM1 | DM vs non- DM | 4 | 868/495012 | 1.65 (1.30-2.10) | 1.83 (1.21-2.78) | 1.38 (1.01-1.89) | 4.62E-05 | 0.0045 | 0.36-9.32 |
| Zhang 2017 | DM2 | DM vs non- DM | 13 | 4168/2373203 | 1.20 (1.15-1.26) | 1.24 (1.06-1.44) | 1.23 (1.15-1.32) | 2.37E-14 | 6.60E-03 | 0.76-2.02 |
| **GDM** | | | | | | | | | | |
| Wang 2020 | GDM | GDM vs non- GDM | 3 | 63694/1163875 | 1.02 (0.81-1.29) | 1.02 (0.81-1.29) | 0.90 (0.65-1.26) | 0.84 | 0.84 | 0.23-4.49 |
| Wang 2020 | GDM | GDM vs non- GDM | 1 | 34294/68588 | 0.31 (0.07-1.42) | 0.31 (0.07-1.42) | 0.31 (0.07-1.46) | 0.13 | 0.13 | N/A |
| Wang 2020 | GDM | GDM vs non- GDM | 4 | 70263/1266775 | 1.12 (0.93-1.34) | 1.14 (0.90-1.44) | 0.96 (0.72-1.28) | 0.24 | 0.28 | 0.53-2.45 |
| **Obstetric, maternal** | | | | | | | | | | |
| **DM** | | | | | | | | | | |
| Inkster 2006 | DM 1/2 | Poor vs optimal glycaemic control | 3 | 85/544 | 2.14 (1.13-4.04) | 2.14 (1.13-4.04) | 1.77 (0.88-3.72) | 1.93E-02 | 1.93E-02 | 0.03-131.76 |
| **GDM** | | | | | | | | | | |
| Wendland 2012 | GDM (WHO criteria) | GDM vs non- GDM | 4 | 5800/30045 | 1.38 (1.29-1.47) | 1.37 (1.25-1.50) | 1.42 (1.31-1.53) | 1.80E-22 | 1.08E-11 | 1.03-1.81 |
| Wendland 2012 | GDM (IADPSG-criteria) | GDM vs non- GDM | 3 | 1909/35052 | 1.80 (1.64-1.99) | 1.71 (1.37-2.13) | 2.02 (1.78-2.29) | 3.07E-33 | 2.45E-06 | 0.14-21.04 |
| Wendland 2012 | GDM (WHO criteria) | GDM vs non- GDM | 3 | 1301/26677 | 1.61 (1.40-1.85) | 1.70 (1.31-2.20) | 1.55 (1.33-1.81) | 3.61E-11 | 6.27E-05 | 0.13-22.01 |
| Wilson 2019 | GDM | GDM vs non- GDM | 12 | 2083/1064553 | 1.12 (1.07-1.18) | 1.54 (1.22-1.95) | 1.06 (1.00-1.11) | 4.45E-06 | 3.03E-04 | 0.74-3.22 |
| Wilson 2019 | GDM | GDM vs non- GDM | 2 | 208/2152 | 1.87 (1.03-3.39) | 1.87 (1.03-3.39) | 1.69 (0.88-3.23) | 0.039 | 0.039 | N/A |
| Manerkar 2020 | GDM | GDM vs non- GDM | 2 | 58/272 | (-)0.36 (-0.67--0.04) | (-)0.36 (-0.67--0.04) | (-)0.45 (-0.86--0.05) | 0.025 | 0.025 | N/A |
| Manerkar 2020 | GDM | GDM vs non- GDM | 5 | 901/9716 | (-)0.19 (-0.26--0.12) | (-)0.24 (-0.42--0.07) | (-)0.07 (-0.17-0.03) | 2.31E-07 | 0.0073 | (-)0.84-0.35 |
| Wendland 2012 | GDM (IADPSG-criteria) | GDM vs non- GDM | 3 | 6828/33788 | 1.28 (1.22-1.35) | 1.23 (0.99-1.53) | 1.45 (1.36-1.55) | 4.25E-24 | 6.58E-02 | 0.08-19.86 |
| Wilson 2019 | GDM | GDM vs non- GDM | 4 | 47/639 | 1.16 (0.74-1.83) | 1.31 (0.70-2.46) | 0.86 (0.48-1.56) | 0.52 | 0.4 | 0.15-11.12 |
| Manerkar 2020 | GDM | GDM vs non- GDM | 2 | 58/272 | 0.16 (-0.16-0.47) | 0.03 (-1.15-1.21) | 0.62 (0.22-1.03) | 0.34 | 0.96 | N/A |
| Manerkar 2020 | GDM | GDM vs non- GDM | 2 | 58/272 | (-)0.13 (-0.45-0.18) | (-)0.13 (-0.45-0.18) | (-)0.17 (-0.57-0.24) | 0.42 | 0.42 | N/A |
| Manerkar 2020 | GDM | GDM vs non- GDM | 2 | 58/272 | (-)0.10 (-0.42-0.21) | (-)0.13 (-0.61-0.35) | 0.09 (-0.32-0.49) | 0.53 | 0.59 | N/A |
| **Obstetric, fetal** | | | | | | | | | | |
| **DM** | | | | | | | | | | |
| Zhao 2015 | PGDM | PGDM vs non- DM | 13 | 33400/1533451 | 2.48 (2.26-2.71) | 2.43 (1.92-3.09) | 3.57 (3.00-4.25) | <1.00E-100 | 1.99E-13 | 1.13-5.23 |
| Chen 2019 | PGDM | PGDM vs non- DM | 13 | 66967/6974271 | 3.93 (3.71-4.17) | 3.41 (2.89-4.03) | 4.36 (4.02-4.73) | <1.0E-100 | <1.0E-100 | 2.02-5.77 |
| Flenady 2011 | PGDM | PGDM vs non- DM | 3 | 3614/564896 | 2.36 (1.71-3.25) | 2.52 (1.52-4.17) | 1.82 (1.16-2.84) | 1.97E-07 | 3.40E-04 | 0.01-530.07 |
| Simeone 2015 | PGDM | PGDM vs non- DM | 8 | 173/1281197 | 3.59 (3.07-4.20) | 3.64 (2.73-4.85) | 3.4 (2.6-4.5) | <1.00E-100 | 1.07E-18 | 1.61-8.22 |
| Inkster 2006 | DM 1/2 | Poor vs optimal glycaemic control | 8 | 238/3684 | 2.33 (1.60-3.40) | 2.33 (1.60-3.40) | 1.90 (1.19-3.11) | 1.05E-05 | 1.05E-05 | 1.46-3.73 |
| Inkster 2006 | DM 1/2 | Poor vs optimal glycaemic control | 4 | 95/797 | 4.53 (1.61-12.77) | 4.53 (1.61-12.77) | 4.06 (1.12-22.12) | 4.22E-03 | 4.22E-03 | 0.47-44.01 |
| Inkster 2006 | DM 1/2 | Poor vs optimal glycaemic control | 3 | 72/2701 | 2.75 (1.46-5.17) | 2.75 (1.46-5.17) | 2.73 (1.46-5.49) | 1.66E-03 | 1.66E-03 | 0.05-164.28 |
| Balsells 2009 | DM 1/2 | DM2 vs DM1 | 22 | 279/8797 | 1.51 (1.16-1.97) | 1.51 (1.16-1.97) | 1.10 (0.68-1.77) | 2.41E-03 | 2.41E-03 | 1.14-2.00 |
| Balsells 2009 | DM 1/2 | DM2 vs DM1 | 16 | 74/6531 | 1.59 (0.96-2.64) | 1.59 (0.96-2.64) | 0.99 (0.38-2.54) | 7.23E-02 | 7.23E-02 | 0.91-2.80 |
| Balsells 2009 | DM 1/2 | DM2 vs DM1 | 19 | 168/7407 | 1.21 (0.85-1.72) | 1.23 (0.82-1.85) | 1.14 (0.66-1.96) | 0.29 | 0.31 | 0.62-2.46 |
| Balsells 2009 | DM 1/2 | DM2 vs DM1 | 24 | 514/9693 | 1.16 (0.94-1.42) | 1.19 (0.91-1.56) | 0.90 (0.58-1.40) | 0.18 | 0.20 | 0.59-2.41 |
| **GDM** | | | | | | | | | | |
| Wendland 2012 | GDM (WHO criteria) | GDM vs non- GDM | 4 | 2755/28755 | 1.53 (1.39-1.68) | 1.53 (1.39-1.68) | 1.51 (1.36-1.68) | 1.10E-17 | 1.10E-17 | 1.24-1.89 |
| Zhao 2015 | GDM | GDM vs non- GDM | 17 | 41668/1816289 | 1.19 (1.11-1.27) | 1.18 (1.08-1.28) | 1.18 (1.07-1.30) | 4.39E-07 | 1.39E-04 | 1.02-1.36 |
| Wendland 2012 | GDM (IADPSG criteria) | GDM vs non- GDM | 3 | 3392/35902 | 1.90 (1.77-2.03) | 1.75 (1.39-2.20) | 1.95 (1.79-2.13) | <1.0E-100 | 1.51E-06 | 0.11-28.60 |
| Manerkar 2020 | GDM | GDM vs non- GDM | 5 | 19594/29089 | 1.36 (1.22-1.52) | 1.49 (1.18-1.88) | 1.33 (1.17-1.52) | 2.24E-08 | 0.00081 | 0.75-2.96 |
| Wendland 2012 | GDM (WHO criteria) | GDM vs non- GDM | 5 | 804/11588 | 1.81 (1.47-2.22) | 1.81 (1.47-2.22) | 1.66 (1.29-2.13) | 2.12E-08 | 2.12E-08 | 1.29-2.53 |
| Van der Looven 2019 | GDM | GDM vs non- GDM | 3 | 244/54484 | 3.94 (2.70-5.76) | 3.94 (2.70-5.76) | 3.28 (2.00-5.39) | 1.23E-12 | 1.23E-12 | 0.34-45.86 |
| Tabrizi 2019 | GDM in Iranian women | GDM vs non GDM | 3 | 103/2491 | 3.79 (2.34-6.13) | 3.77 (1.97-7.21) | 2.50 (1.16-5.39) | 5.53E-08 | 5.99E-05 | 0.01-2276 |
| Li 2019 | GDM | GDM vs non -GDM | 9 | 12659/1609618 | 1.41 (1.28-1.54) | 1.44 (1.16-1.78) | 1.50 (1.30-1.70) | 2.23E-13 | 0.0011 | 0.76-2.71 |
| Zhang 2015 | GDM | GDM vs non- GDM | 6 | 8212/180374 | 1.02 (0.90-1.15) | 1.04 (0.86-1.27) | 1.07 (0.90-1.27) | 0.77 | 0.66 | 0.68-1.60 |
| Wendland 2012 | GDM (WHO criteria) | GDM vs non- GDM | 2 | 127/9072 | 1.55 (0.88-2.73) | 1.55 (0.88-2.73) | 1.48 (0.82-2.66) | 0.13 | 0.13 | N/A |
| **PGDM and GDM** | | | | | | | | | | |
| Li 2019 | DM (PGDM and GDM) | DM vs non- DM | 20 | 81395/2028020 | 1.24 (1.17-1.32) | 1.39 (1.17-1.65) | 1.01 (0.91-1.11) | 1.70E-13 | 1.73E-04 | 0.73-2.66 |
| Shu 2019 | PGDM and GDM | DM vs non- DM | 5 | 4470/1937847 | 1.40 (1.27-1.55) | 1.44 (0.95-2.18) | 1.40 (1.26-1.55) | 6.00E-11 | 0.086 | 0.45-4.66 |

**Abbreviations:** GDM- Gestational diabetes mellitus; PGDM- Pregestational diabetes mellitus; DM 1/2- Diabetes mellitus type 1/2; WHO- World Health Organisation; IADPSG- International Association of the Diabetes and Pregnancy Study Groups

**Key:**

^α^ Number of studies

^β^ Fixed effects refers to summary relative risk 95% CI) using the meta-analysis fixed- effects model

^χ^ Random effects refers to summary relative risk (95% CI) using the meta-analysis random -effects model

^δ^ Relative risk and 95% confidence interval of largest study (smallest SE) in each meta- analysis

^ε^ P value of summary fixed effects estimate

^φ^ P value of summary random effects estimate

^γ^ Prediction intervals are reported only for meta-analyses including at least 3 studies

All statistical tests were two-sided
