# Supplementary material for: Diabetes and anti-diabetic interventions and the risk of gynaecological and obstetric morbidity: an umbrella review of the literature
Source: BMC Med. 2023 Apr 18;21:152. doi: 10.1186/s12916-023-02758-1 (PMC10114404; doi:10.1186/s12916-023-02758-1)
Supplement: Supplementary file 3 — Additional file 3: Table S1B. Evaluation of heterogeneity, small study effects, excess significance bias and credibility ceilings in the 47 meta-analyses investigating the association of diabetes with gynaecological and obstetric morbidity– cohort studies only. [file 12916_2023_2758_MOESM3_ESM.docx]

**Table S1B: Evaluation of heterogeneity, small study effects, excess significance bias and credibility ceilings in the 47 meta-analyses investigating the association of diabetes with gynaecological and obstetric morbidity– cohort studies only**

| **Author, year** | **Exposure** | **Exposure contrast** | **Egger’s P**^α^ | **I^2^ (95% CI) P**^β^ | **Studies** | **Observed**^χ^ | **Expected**^δ^**, P-value**^ε^ | | | | | | **Credibility ceiling (%) p<0.05** |
| --- | --- | --- | --- | --- | --- | --- | --- | --- | --- | --- | --- | --- | --- |
|  |  |  |  |  |  |  | **Fixed**  **effects** | | **Random**  **effects** | | **Largest**  **study** | |  |
| **Gynaecological** | | | | | | | | | | | | | |
| **DM** | | | | | | | | | | | | | |
| Saed 2019 | DM | DM vs non- DM | 0.18 | 69 (23-83) 0.0011 | 9 | 4 | 6.39 | NP | 7.21 | NP | 2.9 | 0.43 | 15 |
| Liao 2014 | DM 1/2 | DM vs non- DM | 0.61 | 0 (0-61) 0.91 | 6 | 1 | 2.24 | NP | 2.24 | NP | 2.26 | NP | 11 |
| Pergaliotis 2016 | DM (unspecified) | DM vs non- DM | 0.49 | 0 (0-56) 0.97 | 8 | 2 | 4.95 | NP | 4.95 | NP | 5.25 | NP | 22 |
| Wang 2017 | DM (unspecified) | DM vs non- DM | 0.38 | 44 (0-69) 0.04 | 14 | 2 | 4.30 | NP | 5.36 | NP | 1.18 | 0.43 | 12 |
| Zhang 2017 | DM 1/2 | DM vs non- DM | 0.47 | 90 (79-94) <1.0E-100 | 5 | 4 | 3.71 | 0.77 | 4.15 | NP | 3.75 | 0.79 | 2 |
| Bhatia 2020 | DM | DM vs non- DM | 0.029 | 0 (0-73) 0.87 | 3 | 0 | 2.86 | NP | 2.86 | NP | 2.73 | NP | 11 |
| Zhang 2017 | DM1 | DM vs non- DM | 0.32 | 56 (0-83) 0.078 | 4 | 3 | 1.5 | 0.12 | 1.66 | 0.17 | 1.29 | 0.068 | 10 |
| Zhang 2017 | DM2 | DM vs non- DM | 0.73 | 82 (69-88) 1.78E-09 | 13 | 3 | 4.92 | NP | 5.73 | NP | 5.59 | NP | 3 |
| **GDM** | | | | | | | | | | | | | |
| Wang 2020 | GDM | GDM vs non- GDM | 0.04 | 0 (0-73) 0.55 | 3 | 0 | 1.14 | NP | 1.14 | NP | 3 | NP | 0 |
| Wang 2020 | GDM | GDM vs non- GDM | N/A | 100 (.-.) <1E-100 | 1 | 0 | 1 | NP | 1 | NP | 1 | NP | 0 |
| Wang 2020 | GDM | GDM vs non- GDM | 0.29 | 32 (0-77) 0.22 | 4 | 1 | 4 | NP | 4 | NP | 2.8 | NP | 0 |
| **Obstetric, maternal** | | | | | | | | | | | | | |
| **DM** | | | | | | | | | | | | | |
| Inkster 2006 | DM 1/2 | Poor vs optimal glycaemic control | 0.50 | 0 (0-73) 0.54 | 3 | 0 | 1.47 | NP | 1.47 | NP | 0.98 | NP | 7 |
| **GDM** | | | | | | | | | | | | | |
| Wendland 2012 | GDM (WHO criteria) | GDM vs non- GDM | 0.92 | 22 (0-75) 0.28 | 4 | 3 | 3.11 | NP | 3.09 | NP | 3.18 | NP | 13 |
| Wendland 2012 | GDM (IADPSG criteria) | GDM vs non- GDM | 0.55 | 74 (0-90) 0.022 | 3 | 2 | 2.94 | NP | 2.89 | NP | 2.99 | NP | 11 |
| Wendland 2012 | GDM (WHO criteria) | GDM vs non- GDM | 0.58 | 40 (0-82) 0.19 | 3 | 2 | 2.30 | NP | 2.46 | NP | 2.18 | NP | 11 |
| Wilson 2019 | GDM | GDM vs non- GDM | 0.006 | 79 (61-86) 3.22E-07 | 12 | 7 | 2.41 | 9.35E-04 | 5.35 | 0.34 | 1.8 | 2.70E-05 | 15 |
| Wilson 2019 | GDM | GDM vs non- GDM | N/A | 0 (.-.) 0.44 | 2 | 0 | 1.11 | NP | 1.11 | NP | 1.07 | NP | 6 |
| Manerkar 2020 | GDM | GDM vs non- GDM | N/A | 0 (.-.) 0.48 | 2 | 1 | 1.79 | NP | 1.79 | NP | 1.93 | NP | 3 |
| Manerkar 2020 | GDM | GDM vs non- GDM | 0.46 | 77 (17-89) 0.0019 | 5 | 3 | 4.37 | NP | 4.58 | NP | 2.82 | 0.87 | 12 |
| Wendland 2012 | GDM (IADPSG-criteria) | GDM vs non- GDM | 0.51 | 95 (88-97) <1.0E-100 | 3 | 2 | 3.00 | NP | 2.96 | NP | 3.00 | NP | 0 |
| Wilson 2019 | GDM | GDM vs non- GDM | 0.25 | 34 (0-78) 0.21 | 4 | 1 | 0.23 | 0.1 | 0.31 | 0.2 | 0.23 | 0.1 | 0 |
| Manerkar 2020 | GDM | GDM vs non- GDM | N/A | 92 (.-.) 0.0003 | 2 | 2 | 0.83 | 0.09 | 0.13 | <1E-100 | 2 | NP | 0 |
| Manerkar 2020 | GDM | GDM vs non- GDM | N/A | 0 (.-.) 0.76 | 2 | 0 | 0.64 | NP | 0.64 | NP | 0.95 | NP | 0 |
| Manerkar 2020 | GDM | GDM vs non- GDM | N/A | 55 (.-.) 0.14 | 2 | 0 | 0.43 | NP | 0.64 | NP | 0.36 | NP | 0 |
| **Obstetric, fetal** | | | | | | | | | | | | | |
| **DM** | | | | | | | | | | | | | |
| Zhao 2015 | PGDM | PGDM vs non- DM | 0.94 | 78 (60-86) <1.0E-100 | 13 | 9 | 10.6 | NP | 10.6 | NP | 11.2 | NP | 27 |
| Chen 2019 | PGDM | PGDM vs non- DM | 0.058 | 76 (56-85) 9.64E-07 | 13 | 10 | 12.9 | NP | 12.9 | NP | 13 | NP | 22 |
| Flenady 2011 | PGDM | PGDM vs non- DM | 0.36 | 56 (0-86) 0.11 | 3 | 3 | 3.00 | NP | 3.00 | NP | 2.95 | 0.83 | 11 |
| Simeone 2015 | PGDM | PGDM vs non- DM | 0.97 | 61 (0-80) 0.013 | 8 | 6 | 5.77 | 0.857 | 5.81 | 0.879 | 5.60 | 0.749 | 16 |
| Inkster 2006 | DM 1/2 | Poor vs optimal glycaemic control | 0.01 | 0 (0-56) 0.82 | 8 | 2 | 4.06 | NP | 4.06 | NP | 3.01 | NP | 22 |
| Inkster 2006 | DM 1/2 | Poor vs optimal glycaemic control | 0.22 | 0 (0-68) 0.96 | 4 | 0 | 3.3 | NP | 3.3 | NP | 3.16 | NP | 15 |
| Inkster 2006 | DM 1/2 | Poor vs optimal glycaemic control | 0.73 | 0 (0-73) 0.96 | 3 | 1 | 1.28 | NP | 1.28 | NP | 1.27 | NP | 4 |
| Balsells 2009 | DM 1/2 | DM2 vs DM1 | 0.43 | 0 (0-41) 0.52 | 22 | 2 | 2.67 | NP | 2.67 | NP | 1.14 | 0.40 | 10 |
| Balsells 2009 | DM 1/2 | DM2 vs DM1 | 0.08 | 0 (0-47) 0.80 | 16 | 1 | 1.26 | NP | 1.26 | NP | 0.70 | 0.71 | 0 |
| Balsells 2009 | DM 1/2 | DM2 vs DM1 | 0.64 | 8 (0-48) 0.36 | 19 | 1 | 1.11 | NP | 1.16 | NP | 1.00 | NP | 0 |
| Balsells 2009 | DM 1/2 | DM2 vs DM1 | 0.45 | 25 (0-54) 0.13 | 24 | 1 | 1.56 | NP | 1.73 | NP | 1.39 | NP | 0 |
| **GDM** | | | | | | | | | | | | | |
| Wendland 2012 | GDM (WHO criteria) | GDM vs non- GDM | 0.478 | 0 (0-68) 0.94 | 4 | 2 | 2.92 | NP | 2.92 | NP | 2.88 | NP | 15 |
| Zhao 2015 | GDM | GDM vs non- GDM | 0.97 | 10 (0-52) 0.34 | 17 | 3 | 5.72 | NP | 5.58 | NP | 5.61 | NP | 12 |
| Wendland 2012 | GDM (IADPSG criteria) | GDM vs non- GDM | 0.465 | 87 (48-94) 0.003 | 3 | 2 | 3.00 | NP | 3.00 | NP | 3.00 | NP | 9 |
| Manerkar 2020 | GDM | GDM vs non- GDM | 0.14 | 56 (0-82) 0.057 | 5 | 3 | 3.67 | NP | 3.97 | NP | 3.58 | NP | 14 |
| Wendland 2012 | GDM (WHO criteria) | GDM vs non- GDM | 0.504 | 0 (0-64) 0.60 | 5 | 3 | 3.39 | NP | 3.39 | NP | 3.07 | NP | 14 |
| Van der Looven 2019 | GDM | GDM vs non- GDM | 0.067 | 0 (0-73) 0.47 | 3 | 3 | 3 | NP | 3 | NP | 3 | NP | 12 |
| Tabrizi 2019 | GDM in Iranian women | GDM vs non GDM | 0.91 | 44 (0-83) 0.17 | 3 | 3 | 2.95 | 0.82 | 2.95 | 0.82 | 2.44 | 0.41 | 11 |
| Li 2019 | GDM | GDM vs non- GDM | 0.69 | 71 (31-84) 0.00052 | 9 | 5 | 6.66 | NP | 6.77 | NP | 6.96 | NP | 9 |
| Zhang 2015 | GDM | GDM vs non- GDM | 0.12 | 28 (0-71) 0.22 | 6 | 0 | 0.39 | NP | 0.81 | NP | 1.45 | NP | 0 |
| Wendland 2012 | GDM (WHO criteria) | GDM vs non- GDM | N/A | 0 (.-.) 0.56 | 2 | 0 | 0.87 | NP | 0.87 | NP | 0.75 | NP | 0 |
| **PGDM and GDM** | | | | | | | | | | | | | |
| Li 2019 | DM (PGDM and GDM) | DM vs non- DM | 0.20 | 81 (72-87) 2.78E-13 | 20 | 8 | 11.9 | NP | 14 | NP | 1.19 | <1E-100 | 8 |
| Shu 2019 | PGDM and GDM | DM vs non- DM | 0.65 | 49 (0-80) 0.098 | 5 | 2 | 2.97 | NP | 3.02 | NP | 2.97 | NP | 0 |

**Abbreviations:** GDM- Gestational diabetes mellitus; PGDM- Pregestational diabetes mellitus; DM 1/2- Diabetes mellitus type 1,2; WHO- World Health Organisation; IADPSG- International Association of the Diabetes and Pregnancy Study Groups; NP- not pertinent (because the estimated is larger than the observed, and there is no evidence of excess statistical significance based on the assumption made for the plausible effect size)

**Key:**

^α^ P-value from the Egger’s regression asymmetry test (P<0.10)

^β^ I^2^ metric of inconsistency (95% confidence interval) and the P-value of the Q test

^χ^ Observed number of statistically significant studies in each meta-analysis

^δ^ Expected number of statistically significant studies using the point estimate of each meta-analysis (from fixed effect, random effect of largest study accordingly) as the plausible effect size

^ε^P value of the excess statistical significance test

All statistical tests were two-sided
