# Supplementary material for: Diabetes and anti-diabetic interventions and the risk of gynaecological and obstetric morbidity: an umbrella review of the literature
Source: BMC Med. 2023 Apr 18;21:152. doi: 10.1186/s12916-023-02758-1 (PMC10114404; doi:10.1186/s12916-023-02758-1)
Supplement: Supplementary file 4 — Additional file 4: Table S2. AMSTAR 2 methodological quality assessment for observational systematic reviews investigating the association of diabetes and anti-diabetic interventions with gynaecological and obstetric morbidity- cohorts only. [file 12916_2023_2758_MOESM4_ESM.docx]

**Table S2. AMSTAR 2 methodological quality assessment for observational systematic reviews investigating the association of diabetes and anti-diabetic interventions with gynaecological and obstetric morbidity- cohorts only**

| **AMSTAR 2 Questions**  **Study Author, year** | PICO | ‘A priori ‘ design and deviations justified | Study design | Literature search | Duplicate study selection review | Duplicate data extraction | Excluded studies | Description of included studies | Assess risk of bias | Funding | Statistical methods for meta-analysis | Impact of RoB from meta-analysis | RoB in individual studies in results | Heterogeneity | Small study bias | Conflict of interest | **Score** |
| --- | --- | --- | --- | --- | --- | --- | --- | --- | --- | --- | --- | --- | --- | --- | --- | --- | --- |
| **Observational Studies** | | | | | | | | | | | | | | | | | |
| **Balsells 2009** | ● | ○ | ○ | ◎ | ● | ○ | ○ | ◎ | ● | ○ | ● | ○ | ○ | ○ | ● | ● | Critically low |
| **Bhatia 2020** | ● | ● | ○ | ◎ | ● | ○ | ○ | ● | ● | ○ | ● | ○ | ● | ● | ● | ● | Low |
| **Chen 2019** | ● | ○ | ○ | ◎ | ○ | ○ | ○ | ◎ | ● | ○ | ● | ○ | ● | ● | ● | ● | Critically low |
| **Flenady 2011** | ● | ○ | ○ | ◎ | ● | ● | ○ | ● | ● | ○ | ● | ● | ● | ○ | ○ | ● | Critically low |
| **Inkster 2006** | ● | ○ | ○ | ◎ | ○ | ○ | ○ | ◎ | ● | ○ | ● | ● | ○ | ○ | ● | ● | Critically low |
| **Li 2019** | ● | ○ | ○ | ◎ | ● | ● | ○ | ◎ | ● | ○ | ● | ● | ● | ● | ● | ● | Critically low |
| **Liao 2014** | ● | ○ | ○ | ◎ | ● | ● | ○ | ◎ | ● | ○ | ● | ● | ● | ● | ● | ● | Critically low |
| **Manerkar 2020** | ● | ● | ○ | ● | ● | ● | ● | ● | ● | ○ | ● | ● | ● | ● | ○ | ● | Low |
| **Pergialiotis 2016** | ● | ○ | ○ | ◎ | ● | ○ | ● | ◎ | ● | ○ | ● | ○ | ○ | ○ | ○ | ○ | Critically low |
| **Saed 2019** | ● | ○ | ○ | ◎ | ● | ● | ○ | ◎ | ○ | ○ | ● | ○ | ○ | ● | ● | ● | Critically low |
| **Shu 2019** | ● | ○ | ○ | ◎ | ● | ● | ○ | ● | ● | ○ | ● | ○ | ● | ● | ● | ● | Critically low |
| **Simeone 2015** | ● | ○ | ○ | ◎ | ● | ● | ○ | ● | ○ | ○ | ● | ○ | ● | ○ | ○ | ● | Critically low |
| **Tabrizi 2019** | ● | ○ | ○ | ◎ | ○ | ○ | ○ | ◎ | ● | ○ | ● | ○ | ○ | ● | ● | ● | Critically low |
| **Van der Looven 2019** | ● | ○ | ○ | ◎ | ● | ● | ○ | ● | ● | ○ | ● | ● | ● | ● | ● | ● | Critically low |
| **Wang 2017** | ● | ○ | ○ | ◎ | ● | ● | ○ | ● | ● | ○ | ● | ● | ○ | ○ | ● | ● | Critically low |
| **Wang 2020** | ● | ○ | ○ | ◎ | ● | ● | ○ | ● | ● | ○ | ● | ○ | ○ | ○ | ● | ● | Critically low |
| **Wendland 2012** | ● | ○ | ○ | ◎ | ● | ● | ◎ | ◎ | ● | ○ | ● | ● | ● | ● | ● | ● | Low |
| **Wilson 2019** | ● | ○ | ○ | ◎ | ● | ● | ○ | ● | ● | ○ | ● | ● | ○ | ● | ● | ○ | Critically low |
| **Zhang 2015** | ● | ○ | ○ | ◎ | ○ | ● | ○ | ◎ | ○ | ○ | ● | ○ | ○ | ● | ● | ● | Critically low |
| **Zhang 2017** | ● | ○ | ○ | ◎ | ○ | ○ | ○ | ◎ | ○ | ○ | ● | ○ | ○ | ● | ● | ● | Critically low |
| **Zhao 2015** | ● | ○ | ○ | ◎ | ● | ● | ○ | ◎ | ● | ○ | ● | ● | ○ | ● | ● | ○ | Critically low |
| **Interventional Observational Studies** | | | | | | | | | | | | | | | | | |
| **Alqudah 2018** | ● | ○ | ○ | ● | ● | ○ | ● | ● | ● | ○ | ● | ● | ● | ● | ● | ● | Low |
| **Blanco 2011** | ● | ◎ | ● | ◎ | ● | ○ | ○ | ◎ | ○ | ○ | ○ | ○ | ○ | ○ | ○ | ● | Critically low |
| **Chu 2018** | ● | ○ | ● | ○ | ● | ● | ○ | ● | ● | ○ | ● | ● | ○ | ● | ● | ● | Critically low |
| **Gilbert 2006** | ● | ○ | ● | ○ | ○ | ○ | ● | ○ | ○ | ○ | ○ | ○ | ○ | ○ | ● | ○ | Critically low |
| **Lepercq 2012** | ● | ○ | ○ | ◎ | ● | ○ | ○ | ● | ○ | ○ | ● | ○ | ○ | ● | ○ | ● | Critically low |
| **Lv 2015** | ● | ○ | ● | ○ | ● | ○ | ○ | ○ | ● | ○ | ○ | ○ | ○ | ○ | ○ | ● | Critically low |
| **Pollex 2011** | ● | ○ | ● | ◎ | ● | ● | ○ | ◎ | ○ | ○ | ● | ○ | ○ | ● | ○ | ● | Critically low |
| **Raffone 2019** | ● | ◎ | ○ | ● | ● | ● | ○ | ● | ● | ○ | ● | ○ | ○ | ● | ● | ● | Critically low |
| **Ranasinghe 2015** | ● | ◎ | ○ | ◎ | ● | ○ | ○ | ● | ● | ○ | ● | ○ | ● | ● | ○ | ● | Critically low |
| **Rys 2018** | ● | ○ | ● | ◎ | ● | ○ | ○ | ● | ● | ○ | ○ | ● | ○ | ● | ● | ● | Critically low |
| **Syed 2011** | ● | ○ | ○ | ◎ | ● | ● | ○ | ◎ | ◎ | ○ | ● | ○ | ○ | ○ | ○ | ● | Critically low |
| **Tian 2019** | ● | ○ | ○ | ◎ | ○ | ○ | ○ | ◎ | ● | ○ | ● | ● | ○ | ● | ● | ○ | Critically low |
| **Wahabi 2010** | ● | ○ | ○ | ◎ | ● | ● | ● | ● | ● | ○ | ● | ● | ● | ● | ○ | ○ | Critically low |
| **Waugh 2010** | ● | ○ | ○ | ◎ | ● | ● | ○ | ● | ● | ● | ● | ● | ● | ● | ● | ● | Critically low |
| **Wen 2019** | ○ | ◎ | ○ | ○ | ○ | ● | ○ | ◎ | ○ | ○ | ● | ○ | ● | ● | ● | ● | Critically low |
| **Zheng 2015** | ● | ○ | ○ | ◎ | ● | ● | ○ | ◎ | ● | ○ | ● | ○ | ○ | ● | ○ | ○ | Critically low |

**Abbreviations:** PICO: Patient/Population- Intervention- Comparison- Outcomes, RoB: Risk of Bias

**Key:** ● Yes

◎ Partial yes

○ No

Critical flaw
