# Supplementary material for: Diabetes and anti-diabetic interventions and the risk of gynaecological and obstetric morbidity: an umbrella review of the literature
Source: BMC Med. 2023 Apr 18;21:152. doi: 10.1186/s12916-023-02758-1 (PMC10114404; doi:10.1186/s12916-023-02758-1)
Supplement: Supplementary file 6 — Additional file 6: Table S4. Details of evidence grading for meta-analyses associating diabetes and anti-diabetic interventions with risk of obstetric and gynaecological morbidity– all study types included. [file 12916_2023_2758_MOESM6_ESM.docx]

**Table S4: Details of evidence grading for meta-analyses associating diabetes and anti-diabetic interventions with risk of obstetric and gynaecological morbidity– all study types included**

| **Exposure** | **Exposure contrast** | ***N*^*^** | **Sample size**  **Cases/cohort** | **Largest study^#^** | **Random effects summary**  **RR (95% CI)^¥^** | **Random *P*-value^\|\|^** | **95% Prediction interval** | **Egger’s *P*^∞^** | | **I^2^ (%)** | **Excess significance^§^** | |
| --- | --- | --- | --- | --- | --- | --- | --- | --- | --- | --- | --- | --- |
|  |  |  |  |  |  |  |  |  |  |  | **O/E ^α^** | ***P*-value^ϕ^** |
| **Strong evidence** | | | | | | | | | | | | |
| **Obstetric, maternal** | | | | | | | | | | | | |
| Caesarean section | GDM vs non- GDM | 4 | 5800/30045 | 1.42 (1.31-1.53) | 1.37 (1.25-1.50) | 1.08E-11 | 1.03-1.81 | 0.92 | 22 | | 3/3.18 | NP |
| **Obstetric, fetal** | | | | | | | | | | | | |
| Large for gestational age | GDM vs non- GDM | 4 | 2755/28755 | 1.51 (1.36-1.68) | 1.53 (1.39-1.68) | 1.10E-17 | 1.24-1.89 | 0.478 | 0 | | 2/2.88 | NP |
| Stillbirth (>20 weeks or  >400 g) | PGDM vs non- DM | 5 | 7680/1892890 | 3.34 (2.46-4.55) | 2.90 (2.05-4.09) | 1.43E-09 | 1.05-7.99 | 0.83 | 49 | | 5/5 | NP |
| **Gynaecological** | | | | | | | | | | | | |
| Cervical ca screening | DM vs non- DM | 14 | 251558/311368 | 0.73 (0.66-0.81) | 0.76 (0.71-0.81) | 2.55E-16 | 0.70-0.81 | 0.94 | 0 | | 4/13.6 | NP |
| **Highly suggestive evidence** | | | | | | | | | | | | |
| **Obstetric, fetal** | | | | | | | | | | | | |
| Major congenital malformations (unspecified) | PGDM vs non- DM | 13 | 33400/1533451 | 3.57 (3.00-4.25) | 2.43 (1.92-3.09) | 1.99E-13 | 1.13-5.23 | 0.94 | 78 | | 9/11.2 | NP |
| Congenital heart defects | PGDM vs non- DM | 31 | 236491/15009001 | 4.36 (4.02-4.73) | 3.18 (2.78-3.65) | <1E-100 | 1.73-5.86 | 0.06 | 79 | | 26/30.8 | NP |
| Brachial plexus palsy | GDM vs non- GDM | 10 | 3209/1651281 | 4.58 (3.34-6.30) | 5.33 (3.77-7.55) | 3.83E-21 | 1.99-14.26 | 0.88 | 59 | | 8/9.69 | NP |
| **Gynaecological** | | | | | | | | | | | | |
| Ovarian ca occurrence | Metformin vs non-metformin, DM2 | 3 | 3288/513702 | 0.16 (0.14-0.17) | 0.18 (0.12-0.25) | 2.52E-23 | 0.01-4.31 | 0.38 | 14 | | 2/2.42 | NP |
| **Suggestive evidence** | | | | | | | | | | | | |
| **Obstetric, maternal** | | | | | | | | | | | | |
| Preeclampsia | GDM vs non- GDM, IADPSG criteria | 3 | 1909/35052 | 2.02 (1.78-2.29) | 1.71 (1.37-2.13) | 2.45E-06 | 0.14-21.04 | 0.55 | 74 | | 2/2.99 | NP |
| Preeclampsia | GDM vs non- GDM,  WHO criteria | 3 | 1301/26677 | 1.55 (1.33-1.81) | 1.70 (1.31-2.20) | 6.27E-05 | 0.13-22.01 | 0.58 | 40 | | 2/2.18 | NP |
| Postnatal depression | GDM vs non- GDM | 15 | 2164/1066123 | 1.06 (1.00-1.11) | 1.59 (1.26-2.00) | 7.53E-05 | 0.74-3.41 | 0.003 | 79 | | 8/1.96 | 3.80E-06 |
| **Obstetric, fetal** | | | | | | | | | | | | |
| Major congenital malformations (unspecified) | GDM vs non- GDM | 17 | 41668/1816289 | 1.18 (1.07-1.30) | 1.18 (1.08-1.28) | 1.39E-04 | 1.02-1.36 | 0.97 | 10 | | 3/5.61 | NP |
| Large for gestational age | GDM vs non- GDM, IADPSG criteria | 3 | 3392/35902 | 1.95 (1.79-2.13) | 1.75 (1.39-2.20) | 1.51E-06 | 0.11-8.60 | 0.465 | 87 | | 2/3.0 | NP |
| Respiratory distress syndrome | GDM vs non- GDM | 13 | 13032/1611494 | 1.50 (1.30-1.70) | 1.57 (1.28-1.93) | 2.00E-05 | 0.82-3.03 | 0.36 | 71 | | 8/8.68 | NP |
| Introduction of formula milk/breastmilk substitute before hospital discharge | GDM vs non- GDM | 5 | 19594/29089 | 1.33 (1.17-1.52) | 1.49 (1.18-1.88) | 0.00081 | 0.75-2.96 | 0.14 | 56 | | 3/3.58 | NP |
| Respiratory distress syndrome | DM vs non- DM | 24 | 81768/2029896 | 1.01 (0.91-1.11) | 1.47 (1.24-1.74) | 6.34E-06 | 0.75-2.89 | 0.09 | 81 | | 11/1.4 | <1E-100 |
| **Gynaecological** | | | | | | | | | | | | |
| Endometrial cancer incidence | DM vs non- DM | 9 | 3564/429206 | 1.16 (0.90-1.48) | 1.56 (1.21-2.01) | 5.04E-04 | 0.72-3.37 | 0.18 | 69 | | 4/2.9 | 0.43 |
| Endometrial cancer survival | Metformin vs other anti-diabetics | 3 | 1368/2015 | 0.43 (0.24-0.77) | 0.47 (0.33-0.67) | 3.84E-05 | 0.04-4.88 | 0.19 | 0 | | 2/2.99 | NP |
| **Weak evidence** | | | | | | | | | | | | |
| **Obstetric, maternal** | | | | | | | | | | | | |
| Antenatal depression | GDM vs non- GDM | 6 | 358/4387 | 1.52 (1.09-2.12) | 2.08 (1.42-3.05) | 0.00018 | 0.77-5.61 | 0.41 | 46 | | 2/2 | NP |
| Duration of breastfeeding | GDM vs non- GDM | 5 | 901/9716 | (-)0.07 (-0.17-0.03) | (-)0.24 (-0.42--0.07) | 0.0073 | (-)0.84-0.35 | 0.46 | 77 | | 3/2.82 | 0.87 |
| Breastmilk protein content | GDM vs non- GDM | 2 | 58/272 | (-)0.45 (-0.86--0.05) | (-)0.36 (-0.67--0.04) | 0.025 | N/A | N/A | 44 | | 1/1.93 | NP |
| Miscarriage | Poor vs optimal glycaemic control (DM 1/2) | 4 | 126/1117 | 1.77 (0.88-3.72) | 3.15 (1.61-6.14) | 7.72E-04 | 0.37-26.86 | 0.71 | 28 | | 1/1.47 | NP |
| Miscarriage | Continuous sc Ins infusion vs Multiple daily inj (DM1) | 14 | 476/4257 | 2.15 (1.59-2.91) | 1.74 (1.37-2.20) | 4.91E-06 | 1.04-2.91 | 0.17 | 24 | | 4/6.35 | NP |
| **Obstetric, fetal** | | | | | | | | | | | | |
| Macrosomia | GDM vs non- GDM,  WHO criteria | 5 | 804/11588 | 1.66 (1.29-2.13) | 1.81 (1.47-2.22) | 2.12E-08 | 1.29-2.53 | 0.504 | 0 | | 3/3.07 | NP |
| Anorectal malformations | GDM vs non- GDM | 5 | 1100/93772 | 1.18 (0.71-1.98) | 1.81 (1.23-2.65) | 2.54E-03 | 0.68-4.84 | 0.460 | 30 | | 2/1.34 | 0.50 |
| Birth weight | Myonisitol vs placebo (GDM) | 3 | 180/353 | (-)162.00 (-295.08--28.92) | (-)114.52 (-213.67--15.37) | 0.024 | (-)871.93-642.89 | 0.31 | 13 | | 1/ 2.85 | NP |
| Congenital malformations (unspecified) | Poor vs optimal glycaemic control (DM 1/2) | 12 | 306/4943 | 1.90 (1.19-3.11) | 2.96 (2.07-4.22) | 2.05E-09 | 1.77-4.96 | 0.067 | 5 | | 4/4.17 | NP |
| Major congenital malformations | Poor vs optimal glycaemic control (DM 1/2) | 6 | 134/1785 | 3.49 (1.20-10.42) | 5.05 (2.58-9.87) | 2.23E-06 | 1.95-13.05 | 0.520 | 0 | | 2/4.62 | NP |
| Perinatal mortality | Poor vs optimal glycaemic control (DM 1/2) | 4 | 91/3136 | 2.73 (1.46-5.49) | 3.01 (1.75-5.17) | 6.71E-05 | 0.92-9.89 | 0.69 | 0 | | 2/1.93 | 0.94 |
| LGA | Lispro vs Regular Ins (DM1) | 3 | 299/816 | 1.41 (1.06-1.86) | 1.39 (1.14-1.68) | 8.99E-04 | 0.40-4.83 | 0.69 | 0 | | 2/1.03 | 0.24 |
| Macrosomia >4.5kg | Continuous sc Ins infusion vs Multiple daily inj (DM1) | 2 | 27/482 | 2.37 (1.06-5.31) | 2.50 (1.20-5.20) | 0.014 | N/A | N/A | 0 | | 1/0.76 | 0.73 |
| Perinatal mortality | DM2 vs DM1 | 22 | 279/8797 | 1.10 (0.68-1.77) | 1.51 (1.16-1.97) | 2.41E-03 | 1.14-2.00 | 0.43 | 0 | | 2/1.14 | 0.40 |
| Anorectal malformations | PGDM vs non- DM | 4 | 828/53789 | 8.18 (3.86-17.34) | 4.51 (2.55-7.96) | 2.10E-07 | 0.74-27.53 | 0.37 | 27 | | 3/3.99 | NP |
| Congenital malformations | Preconception vs no preconception care (PGDM) | 11 | 124/2361 | 1.60 (0.52-4.93) | 0.29 (0.15-0.56) | 2.31E-04 | 0.06-1.34 | 0.3 | 31 | | 4/1.44 | 0.021 |
| Perinatal mortality | Preconception vs no preconception care (PGDM) | 5 | 33/1015 | 0.28 (0.08-0.96) | 0.36 (0.15-0.87) | 0.023 | 0.08-1.51 | 0.27 | 0 | | 1/1.84 | NP |
| Preterm delivery | Preconception vs no preconception care (PGDM) | 4 | 216/583 | 0.64 (0.47-0.88) | 0.70 (0.55-0.89) | 0.0038 | 0.42-1.19 | 0.49 | 0 | | 1/1.28 | NP |
| Persistent pulmonary hypertension of the newborn | DM vs non- DM | 7 | 4950/1939461 | 1.40 (1.26-1.55) | 1.66 (1.18-2.36) | 0.0041 | 0.67-4.16 | 0.27 | 56 | | 3/4.19 | NP |
| LGA | Lispro vs Regular Ins or NPH (GDM, DM 1/2) | 5 | 372/1071 | 1.50 (1.15-1.96) | 1.42 (1.20-1.68) | 3.47E-05 | 1.08-1.86 | 0.6 | 0 | | 2/1.8 | 0.85 |
| Birth weight | Lispro vs Regular Ins or NPH (GDM, DM 1/2) | 6 | 318/1018 | 128.00 (-23.76-279.76) | 116.44 (28.78-204.11) | 0.0092 | (-)7.74-240.63 | 0.84 | 0 | | 1/3.66 | NP |
| **Gynaecological** | | | | | | | | | | | | |
| Endometrial cancer mortality (disease-specific) | DM 1/2 vs non- DM | 6 | 2075/1,268,756 | 1.33 (1.07-1.65 | 1.32 (1.11-1.57) | 1.6E-3 | 1.03-1.69 | 0.61 | 0 | | 1/2.26 | NP |
| Ovarian cancer mortality (disease-specific) | DM 1/2 vs non- DM | 5 | 15312/610592 | 1.32 (1.16-1.5) | 1.44 (1.08-1.93) | 1.35E-02 | 0.49-4.25 | 0.47 | 90 | | 4/3.75 | 0.79 |
| Ovarian cancer incidence | DM1 vs non- DM | 4 | 868/495012 | 1.38 (1.01-1.89) | 1.83 (1.21-2.78) | 0.0045 | 0.36-9.32 | 0.32 | 56 | | 3/1.29 | 0.068 |
| Hirsutism prevalence | DM1 vs non- DM | 8 | 120/471 | 0.38 (0.27-0.5) | 0.29 (0.21-0.40) | 6.10E-14 | 0.11-0.76 | 0.14 | 71 | | 7/3.24 | 0.0068 |
| Hyperandrogenemia prevalence | DM1 vs non- DM | 6 | 88/383 | 0.45 (0.31-0.59) | 0.27 (0.19-0.37) | 1.20E-14 | 0.09-0.76 | 0.36 | 67 | | 6/1.86 | 2.63E-04 |
| Menstrual dysfunction prevalence | DM1 vs non- DM | 8 | 117/473 | 0.28 (0.20-0.37) | 0.25 (0.19-0.34) | 9.28E-21 | 0.11-0.56 | 0.11 | 56 | | 8/4.48 | 0.012 |
| PCOM prevalence | DM1 vs non- DM | 7 | 105/343 | 0.55 (0.39-0.70) | 0.35 (0.26-0.47) | 1.09E-12 | 0.15-0.83 | 0.29 | 65 | | 7/1.32 | 4.00E-08 |
| PCOS prevalence | DM1 vs non- DM | 8 | 99/413 | 0.37 (0.28-0.46) | 0.25 (0.17-0.34) | 3.13E-16 | 0.09-0.64 | 0.02 | 63 | | 8/2.85 | 1.41E-04 |
| Ovarian cancer incidence | DM2 vs non- DM | 13 | 4168/2373203 | 1.23 (1.15-1.32) | 1.24 (1.06-1.44) | 6.60E-03 | 0.76-2.02 | 0.73 | 82 | | 3/5.59 | NP |
| Cervical cancer occurrence | Metformin vs non- metformin (DM2) | 2 | 481/144262 | 0.60 (0.43-0.84) | 0.60 (0.43-0.83) | 0.0023 | N/A | N/A | 0 | | 1/1.1 | NP |
| Premalignant/Malignant endometrial polyps | DM vs non- DM | 8 | 154/5212 | 3.04 (1.02-9.05) | 2.85 (1.72-4.72) | 4.53E-05 | 1.52-5.35 | 0.49 | 0 | | 2/5.25 | NP |
| Ovarian cancer incidence | DM vs non- DM | 14 | 5534/3708313 | 1.05 (0.93-1.2) | 1.19 (1.06-1.34) | 4.36E-03 | 0.87-1.62 | 0.38 | 44 | | 2/1.18 | 0.43 |

**Abbreviations:** GDM- Gestational diabetes mellitus; PGDM- Pregestational diabetes mellitus; DM 1/2- Diabetes mellitus type 1/2; ca- cancer; PCOM- Polycystic ovary morphology; PCOS- Polycystic ovary syndrome; WHO- World Health Organisation; IADPSG- International Association of the Diabetes and Pregnancy Study Groups; NP- not pertinent (because the estimated is larger than the observed, and there is no evidence of excess statistical significance based on the assumption made for the plausible effect size)

**Key:**

*only meta-analyses meeting at least weak grade of evidence listed

^*^ Number of studies

^#^ Relative risk and 95% confidence interval of largest study (smallest standard error) in each meta-analysis

**^¥^** Random effects refer to summary risk ratio (95% confidence interval) using the random-effects model

^||^ *P* value of summary random effects estimate

^∞^ *P*-value from the Egger’s regression asymmetry test

^§^ Expected number of statistically significant studies using the point estimate of the largest study (smallest standard error) as the plausible effect size

^α^ Observed/Expected number of statistically significant studies

ϕ*P* value of the excess statistical significance test

All statistical tests were two-sided

^¶^Small study effect is based on the *P*-value from the Egger’s regression asymmetry test (*P*>0.1) where the random effects summary estimate was larger compared to the point estimate of the largest study in a meta-analysis

†Based on the *p*-value (*P*>0.1) of the excess significance test using the largest study (smallest standard error) in a meta-analysis as the plausible effect size.

Summary of evidence grading criteria:

| Weak | *P*<0.05**^\|\|^** |
| --- | --- |
| Suggestive | *P*<10^-3^**^\|\|^**; >1,000 cases |
| Highly suggestive | *P*<10^-6^**^\|\|^**; >1,000 cases; *P*<0.05 of the largest study in a meta-analysis |
| Strong | *P*<10^-6^**^\|\|^**; >1,000 cases; *P*<0.05 of the largest study in a meta-analysis; I^2^<50%; no small study effect^¶^; prediction interval excludes the null value; no excess significance bias^†^ |
