# Supplementary material for: Diabetes and anti-diabetic interventions and the risk of gynaecological and obstetric morbidity: an umbrella review of the literature
Source: BMC Med. 2023 Apr 18;21:152. doi: 10.1186/s12916-023-02758-1 (PMC10114404; doi:10.1186/s12916-023-02758-1)
Supplement: Supplementary file 7 — Additional file 7: Table S5. Duplicate and excluded meta-analyses and meta-analyses included instead on the effect of diabetes on gynaecological/obstetric outcomes- cohorts only. [file 12916_2023_2758_MOESM7_ESM.docx]

**Table S5: Duplicate and excluded meta-analyses and meta-analyses included instead on the effect of diabetes on gynaecological/obstetric outcomes- cohorts only**

| **Exposure** | **Exposure contrast** | **Author, year** | **Outcome** | **N^a^ studies** | **N cohort studies** | **Summary point estimate & 95% CI^b^** | **Included** | **Evidence grade^c^** |
| --- | --- | --- | --- | --- | --- | --- | --- | --- |
| **Gynaecological outcomes** | | | | | | | | |
| DM | DM vs non- DM | Saed 2019 | Endometrial ca incidence | 9 | 9 | 1.56 (1.21–2.01) | Yes | Suggestive |
| GDM | GDM vs non- GDM | Wang 2020 | Endometrial ca incidence | 2 | 1 | 0.77 (0.20–2.98) | Yes | NS |
| DM 1/2 | DM vs non- DM | Liao 2014 | Endometrial ca incidence | 7 | 7 | 1.71 (1.48- 1.97) | No |  |
| DM (largely type 2) | DM vs non- DM | Zhang 2013 | Endometrial ca incidence | 8 | 8 | 1.92 (1.23- 3.01) | No |  |
| DM (Glycemic index) | Highest vs lowest | Galeone 2013 | Endometrial ca incidence | 7 | 5 | 1.09 (0.92- 1.29) | No |  |
| DM (Glycemic load) | Highest vs lowest | Galeone 2013 | Endometrial ca incidence | 7 | 5 | 1.19 (1.06- 1.34) | No |  |
| DM (Glycemic index) | Highest vs lowest | Choi 2012 | Endometrial ca incidence | 5 | 5 | 1 (0.87- 1.14) | No |  |
| DM (Glycemic load) | Highest vs lowest | Choi 2012 | Endometrial ca incidence | 5 | 5 | 1.21 (1.07- 1.37) | No |  |
| DM | DM vs population | Noto 2010 | Endometrial ca incidence | 4 | 1 | 3.43 (1.53- 7.72) | No |  |
| DM | DM vs population | Friberg 2007 | Endometrial ca incidence | 16 | 3 | 2.1 (1.75- 2.53) | No |  |
| DM 1/2 | DM vs non- DM | Liao 2014 | Endometrial ca mortality (disease-specific) | 6 | 6 | 1.32 (1.10–1.60) | Yes | Weak |
| DM (largely type 2) | DM vs non- DM | Zhang 2013 | Endometrial ca mortality | 3 | 3 | 1.47 (1.06- 2.04) | No |  |
| DM (unspecified) | DM vs non- DM | Wang 2017 | Ovarian ca incidence | 14 | 14 | 1.19 (1.06- 1.34) | Yes | Weak |
| GDM | GDM vs non- GDM | Wang 2020 | Ovarian ca incidence | 4 | 4 | 1.14 (0.90–1.44) | Yes | NS |
| DM | DM vs controls | Lee 2013 | Ovarian ca incidence | 19 | 12 | 1.17 (1.02- 1.33) | No |  |
| DM1 | DM vs non- DM | Zhang 2017 | Ovarian ca incidence | 4 | 4 | 1.83 (1.21- 2.78) | Yes | Weak |
| DM2 | DM vs non- DM | Zhang 2017 | Ovarian ca incidence | 13 | 13 | 1.24 (1.06- 1.44) | Yes | Weak |
| **Maternal obstetric outcomes** | | | | | | | | |
| GDM (WHO criteria) | GDM vs non- GDM | Wendland 2012 | CS | 4 | 4 | 1.37 (1.24-1.51) | Yes | Strong |
| GDM (IADPSG-criteria) | GDM vs non- GDM | Wendland 2012 | CS | 3 | 3 | 1.23 (1.01- 1.51) | Yes | NS |
| PGDM | PGDM vs non- DM | Yu 2017 | CS | 42 | NK | 3.52 (2.91- 4.25) | No |  |
| GDM | GDM vs non- GDM | Farrar 2016 | CS | 4 | 4 | 1.66 (1.52- 1.82) | No |  |
| GDM (IADPSG-criteria) | GDM vs non- GDM | Wendland 2012 | Pre-eclampsia | 3 | 3 | 1.71 (1.37- 2.14) | Yes | Suggestive |
| PGDM | PGDM vs non- DM | Yu 2017 | Pre-eclampsia | 48 | NK | 3.48 (3.01- 4.02) | No |  |
| GDM | GDM vs non- GDM | Wilson 2019 | Postnatal depression | 15 | 12 | 1.59 (1.26, 2.00) | Yes | Suggestive |
| GDM | GDM vs non- GDM | Ross 2016 | Postnatal depression | 6 | NK | NK | No |  |
| PGDM | PGDM vs non- DM | Ross 2016 | Postnatal depression | 5 | NK | NK | No |  |
| **Fetal obstetric outcomes** | | | | | | | | |
| GDM (WHO criteria) | GDM vs non- GDM | Wendland 2012 | LGA | 4 | 4 | 1.53 (1.39- 1.69) | Yes | Strong |
| GDM (IADPSG-criteria) | GDM vs non- GDM | Wendland 2012 | LGA | 3 | 3 | 1.73 (1.28- 2.35) | Yes | Suggestive |
| GDM | GDM vs non- GDM | Roeckner 2016 | LGA | 7 | 7 | 1.12 (0.66- 1.90) | No |  |
| PGDM | PGDM vs non- DM | Zhao 2015 | MCM (unspecified) | 13 | 13 | 2.44 (1.92- 3.10) | Yes | Highly suggestive |
| PGDM | PGDM vs control | Balsells 2012 | MCM | 10 | 9 | 2.66 (2.04- 3.47) | No |  |
| GDM | GDM vs non- GDM | Zhao 2015 | MCM | 17 | 17 | 1.18 (1.11- 1.26) | Yes | Suggestive |
| GDM | GDM vs control | Balsells 2012 | MCM | 17 | 15 | 1.16 (1.07- 1.25) | No |  |
| DM 1/2 | DM2 vs DM1 | Balsells 2009 | MCM | 24 | 24 | 1.19 (0.91- 1.56) | Yes | NS |
| DM 1/2 | Poor vs optimal glycaemic control | Inkster 2006 | MCM | 6 | 4 | 5.14 (2.94- 9.01) | Yes | Weak |
| PGDM | PGDM vs non- DM | Flenady 2011 | Stillbirth (>20 weeks or >400 g) | 5 | 3 | 2.9 (2.05- 4.09) | Yes | Suggestive |
| PGDM | PGDM vs non- DM | Yu 2017 | Stillbirth | 39 | NK | 3.52 (3.19- 3.88) | No |  |
| DM 1/2 | DM2 vs DM1 | Balsells 2009 | Stillbirth | 19 | 19 | 1.23 (0.82- 1.85) | Yes | NS |
| PGDM | PGDM vs non- DM | Chen 2019 | Congenital heart defects | 31 | 13 | 3.18 (2.77–3.65) | Yes | Highly suggestive |
| PGDM | PGDM vs non- DM | Simeone 2015 | Congenital heart defects | 12 | 8 | 3.8 (3.0- 4.9) | No |  |
| Diabetes (PGDM and GDM) | DM vs non- DM | Li 2019 | RDS | 24 | 20 | 1.47 (1.24–1.74) | Yes | Suggestive |
| PGDM | PGDM vs non- DM | Yu 2017 | RDS | NK | NK | 2.05 (1.55- 2.83) | No |  |
| GDM | GDM vs non- GDM | Li 2019 | RDS | 13 | 9 | 2.66 (2.06–3.44) | Yes | Weak |
| GDM (WHO criteria) | GDM vs non- GDM | Wendland 2012 | Macrosomia | 5 | 5 | 1.81 (1.47- 2.22) | Yes | Weak |
| GDM | GDM vs non- GDM | Roeckner 2016 | Macrosomia | 9 | 9 | 0.98 (0.55- 1.76) | No |  |
| GDM | GDM vs non- GDM | He 2015 | Macrosomia (over 4000 / 4500g) | 14 | 4 | 1.71 (1.52- 1.94) | No |  |
| PGDM | PGDM vs non- DM | Yu 2017 | Macrosomia> 4kg | 17 | NK | 1.91(1.74- 2.10) | No |  |
| DM 1/2 | Poor vs optimal glycaemic control | Inkster 2006 | Perinatal mortality | 4 | 3 | 3.03 (1.87- 4.92) | Yes | Weak |
| DM 1/2 | DM2 vs DM1 | Balsells 2009 | Perinatal mortality | 22 | 22 | 1.5 (1.15- 1.96) | Yes | Weak |
| PGDM | PGDM vs non- DM | Yu 2017 | Perinatal mortality | 27 | NK | 3.39 (3.02- 3.81) | No |  |
| GDM (WHO-criteria) | GDM vs non- GDM | Wendland 2012 | Perinatal mortality | 2 | 2 | 1.55 (0.88- 2.73) | Yes | NS |

**Abbreviations**: DM 1/2, diabetes mellitus type 1/2; GDM, gestational diabetes mellitus; PGDM, pregestational diabetes mellitus; WHO, World Health Organisation; IADPSG, International Association of Diabetes and Pregnancy Study Groups; ca, cancer; CS, caesarean section; LGA, large for gestational age; MCM, major congenital malformations; RDS: respiratory distress syndrome; NK, not known; NS, non- significant.

**Key:**

^a^ Number of studies

^b^ Summary relative risk of random effects model including all study types

^c^ Summary of evidence grading criteria:

^d^ P value of summary random effects estimate

^e^ Small study effect is based on the P-value from the Egger’s regression asymmetry test (P>0.1) where the random effects summary estimate was larger compared to the point estimate of the largest study in a meta-analysis

^f^ Based on the p-value (P>0.1) of the excess significance test using the largest study (smallest standard error) in a meta-analysis as the plausible effect size.

| Weak | P<0.05**^d^** |
| --- | --- |
| Suggestive | P<10^-3^**^d^**; >1,000 cases |
| Highly suggestive | P<10^-6^**^d^**; >1,000 cases; P<0.05 of the largest study in a meta-analysis |
| Strong | P<10^-6^**^d^**; >1,000 cases; P<0.05 of the largest study in a meta-analysis; I^2^<50%; no small study effect^e^; prediction interval excludes the null value; no excess significance bias^f^ |
