# Supplementary material for: Diabetes and anti-diabetic interventions and the risk of gynaecological and obstetric morbidity: an umbrella review of the literature
Source: BMC Med. 2023 Apr 18;21:152. doi: 10.1186/s12916-023-02758-1 (PMC10114404; doi:10.1186/s12916-023-02758-1)
Supplement: Supplementary file 8 — Additional file 8: Table S6A. Description of 49 meta- analyses results investigating the association of anti-diabetic interventions with gynaecological and obstetric morbidity–cohort studies only. [file 12916_2023_2758_MOESM8_ESM.docx]

**Table S6A: Description of 49 meta- analyses results investigating the association of anti-diabetic interventions with gynaecological and obstetric morbidity–cohort studies only**

| **Author, year** | **Exposure** | **Exposure contrast** | **N**^α^ | **Sample size**  **cases/ cohort** | **Summary relative risk (95% CI)** | | | **Fixed**  **P-value**^ε^ | **Random**  **P-value**^φ^ | **95% Prediction interval**^γ^ |
| --- | --- | --- | --- | --- | --- | --- | --- | --- | --- | --- |
|  |  |  |  |  | **Fixed Effects**^β^ | **Random Effects**^χ^ | **Largest Study**^δ^ |  |  |  |
| **Gynaecological** | | | | | | | | | | |
| **DM** | | | | | | | | | | |
| Wen 2019 | Metformin (DM2) | Metformin vs non- metformin | 3 | 3288/513702 | 0.16 (0.15-0.18) | 0.18 (0.12-0.25) | 0.16 (0.14-0.17) | <1.0E-100 | 2.52E-23 | 0.01-4.31 |
| Chu 2018 | Metformin (DM) | Metformin vs other antidiabetic drugs | 3 | 1368/2015 | 0.47 (0.33-0.67) | 0.47 (0.33-0.67) | 0.43 (0.24-0.77) | 3.84E-05 | 3.84E-05 | 0.04-4.88 |
| Wen 2019 | Metformin (DM2) | Metformin vs non- metformin | 2 | 481/144262 | 0.60 (0.43-0.83) | 0.60 (0.43-0.83) | 0.60 (0.43-0.84) | 0.0023 | 0.0023 | N/A |
| Chu 2018 | Metformin (DM) | Metformin vs other antidiabetic drugs | 5 | 5793/5267300 | 0.85 (0.79-0.92) | 1.02 (0.73-1.42) | 0.68 (0.61-0.74) | 2.29E-05 | 0.92 | 0.29-3.60 |
| Wen 2019 | Metformin (DM2) | Metformin vs non- metformin | 4 | 3822/1052116 | 0.31 (0.29-0.33) | 0.71 (0.29-1.73) | 0.23 (0.21-0.25) | <1.0E-100 | 0.45 | 0.01-51.90 |
| Tian 2019 | Anti-diabetic medication (DM) | Metformin vs non- metformin | 3 | 3391/491029 | 0.57 (0.52-0.61) | 1.04 (0.46-2.39) | 0.50 (0.46-0.54) | <1.0E-100 | 0.92 | 0-40828 |
| Raffone 2019 | Conservative Mx of endometrial hyperplasia and cancer (DM) | DM vs non- DM | 2 | 37/404 | 0.96 (0.30-3.07) | 0.96 (0.30-3.07) | 0.79 (0.17-3.60) | 0.95 | 0.95 | N/A |
| Raffone 2019 | Conservative Mx of endometrial hyperplasia and cancer (DM) | DM vs non- DM | 5 | 48/383 | 1.86 (0.61-5.67) | 1.72 (0.43-6.80) | 7.40 (1.18-46.39) | 0.27 | 0.44 | 0.05-56.96 |
| **Obstetric, maternal** | | | | | | | | | | |
| **DM** | | | | | | | | | | |
| Rys 2018 | Insulin (DM1) | Continuous sc Ins infusion vs Multiple daily inj | 10 | 425/3732 | 1.86 (1.55-2.23) | 1.76 (1.39-2.23) | 2.15 (1.59-2.910 | 1.59E-11 | 2.23E-06 | 1.11-2.80 |
| Ranasinghe 2015 | Ins analogs/Regular Ins in MDI arm (DM1) | MDI vs CSII | 3 | 191/293 | 1.08 (0.88-1.33) | 1.11 (0.77-1.58) | 1.14 (0.88-1.48) | 0.45 | 0.58 | 0.02-49.28 |
| Ranasinghe 2015 | Only Ins analogs in MDI arm (DM1) | MDI vs CSII | 3 | 147/220 | 1.01 (0.90-1.14) | 1.01 (0.90-1.14) | 1.00 (0.87-1.14) | 0.82 | 0.82 | 0.47-2.17 |
| Lepercq 2012 | Ins glargine/NPH (DM) | Glargine vs NPH | 3 | 25/330 | 0.43 (0.16-1.19) | 0.52 (0.14-1.97) | 0.24 (0.07-0.87) | 0.1 | 0.34 | 0-90676 |
| Lepercq 2012 | Ins glargine/NPH (DM) | Glargine vs NPH | 7 | 55/672 | 0.64 (0.34-1.19) | 0.47 (0.19-1.20) | 2.34 (0.84-6.51) | 0.16 | 0.11 | 0.04-6.01 |
| Rys 2018 | Insulin (DM1) | Continuous sc Ins infusion vs multiple daily inj | 7 | 148/1125 | 0.91 (0.65-1.27) | 1.13 (0.60-2.15) | 0.76 (0.49-1.16) | 0.57 | 0.71 | 0.20-6.36 |
| Rys 2018 | Insulin (DM1) | Continuous sc Ins infusion vs multiple daily inj | 10 | 313/2741 | 0.77 (0.60-0.99) | 0.94 (0.55-1.62) | 0.92 (0.63-1.34) | 0.038 | 0.84 | 0.18-5.03 |
| **GDM** | | | | | | | | | | |
| Alqudah 2018 | Metformin (GDM) | Metformin vs control | 4 | 49/1404 | 1.17 (0.67-2.04) | 1.22 (0.57-2.62) | 0.91 (0.45-1.84) | 0.58 | 0.62 | 0.10-14.89 |
| **Obstetric, fetal** | | | | | | | | | | |
| **DM** | | | | | | | | | | |
| Wahabi 2010 | Preconception care (PGDM) | Preconception vs no preconception care | 11 | 124/2361 | 0.32 (0.19-0.54) | 0.29 (0.15-0.56) | 1.60 (0.52-4.93) | 1.92E-05 | 2.31E-04 | 0.06-1.34 |
| Wahabi 2010 | Preconception care (PGDM) | Preconception vs no preconception care | 5 | 33/1015 | 0.36 (0.15-0.87) | 0.36 (0.15-0.87) | 0.28 (0.08-0.96) | 0.023 | 0.023 | 0.08-1.51 |
| Wahabi 2010 | Preconception care (PGDM) | Preconception vs no preconception care | 4 | 216/583 | 0.70 (0.55-0.89) | 0.70 (0.55-0.89) | 0.64 (0.47-0.88) | 0.0038 | 0.0038 | 0.42-1.19 |
| Blanco 2011 | Lispro/Regular Ins (DM1) | Lispro vs Regular Ins | 2 | 164/355 | 1.41 (1.09-1.83) | 1.41 (1.09-1.83) | 1.41 (1.06-1.86) | 0.0084 | 0.0084 | N/A |
| Rys 2018 | Insulin (DM1) | Continuous sc Ins infusion vs Multiple daily inj | 2 | 27/482 | 2.50 (1.20-5.20) | 2.50 (1.20-5.20) | 2.37 (1.06-5.31) | 0.014 | 0.014 | N/A |
| Ranasinghe 2015 | Ins (DM1) | MDI vs CSII | 3 | 9/219 | 2.12 (0.38-11.79) | 2.12 (0.38-11.79) | 2.20 (0.26-18.28) | 0.39 | 0.39 | N/A |
| Ranasinghe 2015 | Ins analogs/Regular Ins in MDI arm (DM1) | MDI vs CSII | 3 | 39/306 | 1.18 (0.65-2.17) | 1.18 (0.65-2.17) | 1.20 (0.56-2.58) | 0.58 | 0.58 | 0.02-59.61 |
| Ranasinghe 2015 | Only Ins analogs in MDI arm (DM1) | MDI vs CSII | 3 | 39/220 | 0.97 (0.51-1.84) | 0.97 (0.51-1.84) | 1.15 (0.55-2.40) | 0.92 | 0.92 | 0.02-62.42 |
| Ranasinghe 2015 | Ins analogs/Regular Ins in MDI arm (DM1) | MDI vs CSII | 3 | 50/303 | 1.21 (0.69-2.13) | 1.21 (0.69-2.13) | 1.10 (0.49-2.49) | 0.51 | 0.51 | 0.03-47.48 |
| Ranasinghe 2015 | Only Ins analogs in MDI arm (DM1) | MDI vs CSII | 3 | 61/210 | 0.90 (0.59-1.39) | 0.90 (0.59-1.39) | 0.94 (0.56-1.58) | 0.64 | 0.64 | 0.06-14.53 |
| Lepercq 2012 | Ins glargine/NPH (DM) | Glargine vs NPH | 5 | 40/508 | 0.78 (0.38-1.58) | 0.78 (0.38-1.58) | 1.09 (0.38-3.11) | 0.49 | 0.49 | 0.25-2.46 |
| Lepercq 2012 | Ins glargine/NPH (DM) | Glargine vs NPH | 4 | 76/355 | 1.20 (0.71-2.03) | 1.20 (0.71-2.03) | 1.10 (0.45-2.68) | 0.49 | 0.49 | 0.38-3.80 |
| Lepercq 2012 | Ins glargine/NPH (DM) | Glargine vs NPH | 6 | 117/620 | 0.95 (0.61-1.47) | 0.95 (0.61-1.47) | 1.27 (0.55-2.96) | 0.82 | 0.82 | 0.51-1.77 |
| Lepercq 2012 | Ins glargine/NPH (DM) | Glargine vs NPH | 6 | 188/581 | 0.80 (0.48-1.35) | 0.79 (0.45-1.38) | 1.19 (0.47-2.99) | 0.4 | 0.41 | 0.28-2.22 |
| Rys 2018 | Insulin (DM1) | Continuous sc Ins infusion vs multiple daily inj | 9 | 331/1373 | 0.95 (0.78-1.15) | 0.95 (0.78-1.15) | 0.87 (0.61-1.25) | 0.61 | 0.61 | 0.75-1.20 |
| Rys 2018 | Insulin (DM1) | Continuous sc Ins infusion vs multiple daily inj | 10 | 622/2236 | 0.96 (0.82-1.11) | 0.96 (0.82-1.11) | 1.08 (0.85-1.36) | 0.56 | 0.56 | 0.80-1.14 |
| Rys 2018 | Insulin (DM1) | Continuous sc Ins infusion vs multiple daily inj | 9 | 291/2560 | 0.86 (0.67-1.11) | 0.86 (0.67-1.11) | 0.83 (0.57-1.22) | 0.26 | 0.26 | 0.63-1.17 |
| Rys 2018 | Insulin (DM1) | Continuous sc Ins infusion vs multiple daily inj | 6 | 70/2196 | 1.21 (0.72-2.02) | 1.21 (0.72-2.02) | 1.60 (0.82-3.11) | 0.47 | 0.47 | 0.58-1.51 |
| Gilbert 2006 | Metformin (DM) | Metfomin vs non metformin | 3 | 13/200 | 0.84 (0.14-5.08) | 0.84 (0.14-5.08) | 0.37 (0.02-7.05) | 0.85 | 0.85 | 0-98251 |
| Lv 2015 | Insulin analogs (PGDM) | Glargine vs NPH | 2 | 64/105 | 19.59 (-68.06-107.24) | 19.59 (-68.06-107.24) | 20.00 (-69.25-109.25) | 0.66 | 0.66 | N/A |
| Ranasinghe 2015 | Ins analogs/Regular Ins in MDI arm (DM1) | MDI vs CSII | 2 | 150/244 | (-)24.80 (-245.58-195.99) | (-)24.80 (-245.58-195.99) | (-)2.00 (-265.15-261.15) | 0.83 | 0.83 | N/A |
| Ranasinghe 2015 | Only Ins analogs in MDI arm (DM1) | MDI vs CSII | 3 | 86/220 | 92.22 (-73.15-257.59) | 92.22 (-73.15-257.59) | 147.70 (-96.24-391.64) | 0.27 | 0.27 | (-)979.87-1164.31 |
| **GDM** | | | | | | | | | | |
| Waugh 2010 | Mx for GDM | Glibenclamide vs Insulin | 3 | 14/626 | 3.15 (0.99-9.95) | 3.15 (0.99-9.95) | 3.03 (0.81-11.28) | 0.051 | 0.051 | N/A |
| Waugh 2010 | Mx for GDM | Glibenclamide vs Insulin | 3 | 161/1030 | 1.21 (0.91-1.59) | 1.21 (0.91-1.59) | 1.16 (0.84-1.59) | 0.18 | 0.18 | 0.20-7.28 |
| Waugh 2010 | Mx for GDM | Glibenclamide vs Insulin | 4 | 68/778 | 1.82 (1.08-3.07) | 1.71 (0.58-5.10) | 1.99 (1.00-3.95) | 0.026 | 0.33 | 0.02-175.14 |
| Syed 2011 | Mx for GDM | Optimal vs suboptimal control | 3 | 58/469 | 0.39 (0.24-0.64) | 0.48 (0.21-1.08) | 0.31 (0.17-0.55) | 1.50E-04 | 0.077 | 0-1920 |
| Syed 2011 | Mx for GDM | Optimal vs suboptimal control | 2 | 8/3376 | 0.52 (0.12-2.26) | 0.61 (0.09-4.30) | 0.29 (0.05-1.60) | 0.39 | 0.62 | N/A |
| Zheng 2015 | Myoinositol (GDM) | Myonisitol vs placebo | 2 | 134/270 | (-)106.03 (-216.21-4.16) | (-)88.28 (-260.13-83.57) | (-)162.00 (-295.08--28.92) | 0.059 | 0.31 | N/A |
| **PGDM and GDM** | | | | | | | | | | |
| Lv 2015 | Insulin analogs (GDM, DM 1/2) | Lispro vs Regular Ins or NPH | 4 | 329/875 | 1.42 (1.19-1.70) | 1.42 (1.19-1.70) | 1.50 (1.15-1.96) | 8.45E-05 | 8.45E-05 | 0.97-2.09 |
| Lv 2015 | Insulin analogs (GDM, DM 1/2) | Lispro vs Regular Ins or NPH | 6 | 318/1018 | 116.44 (28.78-204.11) | 116.44 (28.78-204.11) | 128.00 (-23.76-279.76) | 0.0092 | 0.0092 | (-)7.74-240.63 |
| Pollex 2011 | Ins glargine/NPH (PGDM, GDM) | Glargine vs NPH | 5 | 28/325 | 0.97 (0.47-2.01) | 0.97 (0.47-2.01) | 1.08 (0.44-2.63) | 0.94 | 0.94 | 0.20-4.77 |
| Pollex 2011 | Ins glargine/NPH (PGDM, GDM) | Glargine vs NPH | 3 | 51/291 | 1.28 (0.77-2.12) | 1.28 (0.77-2.12) | 1.08 (0.52-2.25) | 0.35 | 0.35 | 0.05-33.99 |
| Pollex 2011 | Ins glargine/NPH (PGDM, GDM) | Glargine vs NPH | 7 | 123/650 | 0.96 (0.69-1.33) | 0.94 (0.64-1.38) | 0.82 (0.45-1.52) | 0.8 | 0.77 | 0.44-2.02 |

**Abbreviations:** GDM- Gestational diabetes mellitus; PGDM- Pregestational diabetes mellitus; DM 1/2- Diabetes mellitus type 1/2; sc-subcutaneous; inj- injections; Ins- Insulin; Mx- Management; NPH- Neutral Protamine Hagedorn; CSII- Continuous subcutaneous insulin infusion; MDI- Multiple daily injections

**Key:**

^α^ Number of studies

^β^ Fixed effects refers to summary relative risk 95% CI) using the meta-analysis fixed- effects model

^χ^ Random effects refers to summary relative risk (95% CI) using the meta-analysis random -effects model.

^δ^ Relative risk and 95% confidence interval of largest study (smallest SE) in each meta- analysis

^ε^ P value of summary fixed effects estimate

^φ^ P value of summary random effects estimate

^γ^ Prediction intervals are reported only for meta-analyses including at least 3 studies

All statistical tests were two-sided
