# Supplementary material for: Diabetes and anti-diabetic interventions and the risk of gynaecological and obstetric morbidity: an umbrella review of the literature
Source: BMC Med. 2023 Apr 18;21:152. doi: 10.1186/s12916-023-02758-1 (PMC10114404; doi:10.1186/s12916-023-02758-1)
Supplement: Supplementary file 9 — Additional file 9: Table S6B. Evaluation of heterogeneity, small study effects, excess significance bias and credibility ceilings in the 49 meta-analyses investigating the association of anti-diabetic interventions with gynaecological and obstetric morbidity– cohort studies only. [file 12916_2023_2758_MOESM9_ESM.docx]

**Table S6B: Evaluation of heterogeneity, small study effects, excess significance bias and credibility ceilings in the 49 meta-analyses investigating the association of anti-diabetic interventions with gynaecological and obstetric morbidity– cohort studies only**

| **Author, year** | **Exposure** | **Exposure contrast** | **Egger’s P**^α^ | **I^2^ (95% CI) P**^β^ | **Studies** | **Observed**^χ^ | **Expected**^δ^**, P-value**^ε^ | | | | | | **Credibility ceiling (%) p<0.05** |
| --- | --- | --- | --- | --- | --- | --- | --- | --- | --- | --- | --- | --- | --- |
|  |  |  |  |  |  |  | **Fixed**  **effects** | | **Random**  **effects** | | **Largest**  **study** | |  |
| **Gynaecological** | | | | | | | | | | | | | |
| **DM** | | | | | | | | | | | | | |
| Wen 2019 | Metformin (DM2) | Metformin vs non- metformin | 0.38 | 14 (0-77) 0.31 | 3 | 2 | 2.41 | NP | 2.38 | NP | 2.42 | NP | 9 |
| Chu 2018 | Metformin (DM) | Metformin vs other antidiabetic drugs | 0.19 | 0 (0-73) 0.69 | 3 | 2 | 2.97 | NP | 2.97 | NP | 2.99 | NP | 12 |
| Wen 2019 | Metformin (DM2) | Metformin vs non- metformin | N/A | 0 (.-.) 0.87 | 2 | 1 | 1.1 | NP | 1.1 | NP | 1.1 | NP | 3 |
| Chu 2018 | Metformin (DM) | Metformin vs other antidiabetic drugs | 0.17 | 93 (87-96) 1.01E-11 | 5 | 3 | 3.5 | NP | 0.31 | 6.20E-07 | 4.98 | NP | 0 |
| Wen 2019 | Metformin (DM2) | Metformin vs non- metformin | 0.14 | 98 (98-99) <1.0E-100 | 4 | 2 | 3.99 | NP | 2.91 | NP | 4 | NP | 0 |
| Tian 2019 | Anti-diabetic medication (DM) | Metformin vs non- metformin | 0.093 | 97 (96-98) 1.22E-17 | 3 | 2 | 2.94 | NP | 0.39 | 0.0056 | 2.99 | NP | 0 |
| Raffone 2019 | Conservative Mx of endometrial hyperplasia and cancer (DM) | DM vs non- DM | N/A | 0 (.-.) 0.70 | 2 | 0 | 0.1 | NP | 0.1 | NP | 0.16 | NP | 0 |
| Raffone 2019 | Conservative Mx of endometrial hyperplasia and cancer (DM) | DM vs non- DM | 0.66 | 29 (0-74) 0.23 | 5 | 1 | 0.81 | 0.82 | 0.67 | 0.66 | 3.9 | NP | 0 |
| **Obstetric, maternal** | | | | | | | | | | | | | |
| **DM** | | | | | | | | | | | | | |
| Rys 2018 | Insulin (DM1) | Continuous sc Ins infusion vs Multiple daily inj | 0.11 | 20 (0-62) 0.26 | 10 | 3 | 4.27 | NP | 3.87 | NP | 5.19 | NP | 7 |
| Ranasinghe 2015 | Ins analogs/Regular Ins in MDI arm (DM1) | MDI vs CSII | 0.77 | 58 (0-86) 0.094 | 3 | 0 | 0.17 | NP | 0.19 | NP | 0.22 | NP | 0 |
| Ranasinghe 2015 | Only Ins analogs in MDI arm (DM1) | MDI vs CSII | 0.86 | 0 (0-73) 0.91 | 3 | 0 | 0.15 | NP | 0.15 | NP | 0.15 | NP | 0 |
| Lepercq 2012 | Ins glargine/NPH (DM) | Glargine vs NPH | 0.28 | 30 (0-80) 0.24 | 3 | 1 | 0.72 | 0.71 | 0.5 | 0.44 | 1.42 | NP | 0 |
| Lepercq 2012 | Ins glargine/NPH (DM) | Glargine vs NPH | 0.023 | 49.9 (0-77) 0.062 | 7 | 1 | 0.7 | 0.7 | 1.33 | NP | 1.61 | NP | 0 |
| Rys 2018 | Insulin (DM1) | Continuous sc Ins infusion vs multiple daily inj | 0.36 | 54 (.-.) 0.04 | 7 | 1 | 0.39 | 0.32 | 0.42 | 0.35 | 0.69 | 0.69 | 0 |
| Rys 2018 | Insulin (DM1) | Continuous sc Ins infusion vs multiple daily inj | 0.29 | 71 (33-83) 0.00035 | 10 | 2 | 1.17 | 0.42 | 0.53 | 0.038 | 0.57 | 0.0499 | 0 |
| **GDM** | | | | | | | | | | | | | |
| Alqudah 2018 | Metformin (GDM) | Metformin vs control | 0.86 | 30 (0-76) 0.23 | 4 | 0 | 0.24 | NP | 0.26 | NP | 0.21 | NP | 0 |
| **Obstetric, fetal** | | | | | | | | | | | | | |
| **DM** | | | | | | | | | | | | | |
| Wahabi 2010 | Preconception care (PGDM) | Preconception vs no preconception care | 0.3 | 31 (0-66) 0.16 | 11 | 4 | 5.1 | NP | 5.58 | NP | 1.44 | 0.021 | 11 |
| Wahabi 2010 | Preconception care (PGDM) | Preconception vs no preconception care | 0.27 | 0 (.-.) 0.93 | 5 | 1 | 1.41 | NP | 1.41 | NP | 1.84 | NP | 5 |
| Wahabi 2010 | Preconception care (PGDM) | Preconception vs no preconception care | 0.49 | 0 (0-68) 0.48 | 4 | 1 | 0.9 | 0.9 | 0.9 | 0.9 | 1.28 | NP | 4 |
| Blanco 2011 | Lispro/Regular Ins (DM1) | Lispro vs Regular Ins | N/A | 0 (.-.) 0.95 | 2 | 1 | 0.58 | 0.52 | 0.58 | 0.52 | 0.57 | 0.51 | 5 |
| Rys 2018 | Insulin (DM1) | Continuous sc Ins infusion vs Multiple daily inj | N/A | 0 (.-.) 0.75 | 2 | 1 | 0.83 | 0.81 | 0.83 | 0.81 | 0.76 | 0.73 | 6 |
| Ranasinghe 2015 | Ins (DM1) | MDI vs CSII | N/A | 0 (.-.) 0.95 | 3 | 0 | 0.27 | NP | 0.27 | NP | 0.29 | NP | 0 |
| Ranasinghe 2015 | Ins analogs/Regular Ins in MDI arm (DM1) | MDI vs CSII | 0.6 | 0 (0-73) 0.99 | 3 | 0 | 0.18 | NP | 0.18 | NP | 0.19 | NP | 0 |
| Ranasinghe 2015 | Only Ins analogs in MDI arm (DM1) | MDI vs CSII | 0.17 | 0 (0-73) 0.56 | 3 | 0 | 0.15 | NP | 0.15 | NP | 0.17 | NP | 0 |
| Ranasinghe 2015 | Ins analogs/Regular Ins in MDI arm (DM1) | MDI vs CSII | 0.27 | 0 (0-73) 0.91 | 3 | 0 | 0.2 | NP | 0.2 | NP | 0.16 | NP | 0 |
| Ranasinghe 2015 | Only Ins analogs in MDI arm (DM1) | MDI vs CSII | 0.054 | 0 (0-73) 0.90 | 3 | 0 | 0.17 | NP | 0.17 | NP | 0.16 | NP | 0 |
| Lepercq 2012 | Ins glargine/NPH (DM) | Glargine vs NPH | 0.82 | 0 (0-64) 0.56 | 5 | 0 | 0.33 | NP | 0.33 | NP | 0.26 | NP | 0 |
| Lepercq 2012 | Ins glargine/NPH (DM) | Glargine vs NPH | 0.25 | 0 (0-68) 0.79 | 4 | 0 | 0.27 | NP | 0.27 | NP | 0.22 | NP | 0 |
| Lepercq 2012 | Ins glargine/NPH (DM) | Glargine vs NPH | 0.032 | 0 (0-61) 0.51 | 6 | 0 | 0.31 | NP | 0.31 | NP | 0.49 | NP | 0 |
| Lepercq 2012 | Ins glargine/NPH (DM) | Glargine vs NPH | 0.48 | 11 (0-65) 0.34 | 6 | 0 | 0.51 | NP | 0.54 | NP | 0.43 | NP | 0 |
| Rys 2018 | Insulin (DM1) | Continuous sc Ins infusion vs multiple daily inj | 0.42 | 0 (0-54) 0.54 | 9 | 0 | 0.47 | NP | 0.47 | NP | 0.63 | NP | 0 |
| Rys 2018 | Insulin (DM1) | Continuous sc Ins infusion vs multiple daily inj | 0.11 | 0 (0-53) 0.54 | 10 | 0 | 0.53 | NP | 0.53 | NP | 0.6 | NP | 0 |
| Rys 2018 | Insulin (DM1) | Continuous sc Ins infusion vs multiple daily inj | 0.63 | 0 (0-54) 0.75 | 9 | 0 | 0.65 | NP | 0.65 | NP | 0.77 | NP | 0 |
| Rys 2018 | Insulin (DM1) | Continuous sc Ins infusion vs multiple daily inj | 0.17 | 0 (0-65) 0.53 | 6 | 0 | 0.39 | NP | 0.39 | NP | 0.84 | NP | 0 |
| Gilbert 2006 | Metformin (DM) | Metfomin vs non- metformin | 0.19 | 0 (0-73) 0.77 | 3 | 0 | 0.16 | NP | 0.16 | NP | 0.57 | NP | 0 |
| Lv 2015 | Insulin analogs (PGDM) | Glargine vs NPH | N/A | 0 (.-.) 0.96 | 2 | 0 | 0.19 | NP | 0.19 | NP | 0.19 | NP | 0 |
| Ranasinghe 2015 | Ins analogs/Regular Ins in MDI arm (DM1) | MDI vs CSII | N/A | 0 (.-.) 0.76 | 2 | 0 | 0.12 | NP | 0.12 | NP | 0.1 | NP | 0 |
| Ranasinghe 2015 | Only Ins analogs in MDI arm (DM1) | MDI vs CSII | 0.75 | 0 (0-73) 0.62 | 3 | 0 | 0.76 | NP | 0.76 | NP | 1.56 | NP | 0 |
| **GDM** | | | | | | | | | | | | | |
| Waugh 2010 | Mx for GDM | Glibenclamide vs Insulin | N/A | 0 (.-.) 0.91 | 3 | 0 | 0.73 | NP | 0.73 | NP | 0.7 | NP | 0 |
| Waugh 2010 | Mx for GDM | Glibenclamide vs Insulin | 0.23 | 0 (0-73) 0.85 | 3 | 0 | 0.32 | NP | 0.32 | NP | 0.26 | NP | 0 |
| Waugh 2010 | Mx for GDM | Glibenclamide vs Insulin | 0.87 | 70 (0-88) 0.017 | 4 | 2 | 0.99 | 0.24 | 0.84 | 0.16 | 1.23 | 0.4 | 0 |
| Syed 2011 | Mx for GDM | Optimal vs suboptimal control | 0.042 | 49.9 (0-84) 0.14 | 3 | 1 | 1.43 | NP | 1.06 | NP | 1.8 | NP | 0 |
| Syed 2011 | Mx for GDM | Optimal vs suboptimal control | N/A | 36 (.-.) 0.21 | 2 | 0 | 0.22 | NP | 0.17 | NP | 0.55 | NP | 0 |
| Zheng 2015 | Myoinositol (GDM) | Myonisitol vs placebo | N/A | 54 (.-.) 0.14 | 2 | 1 | 1.43 | NP | 1.15 | NP | 1.89 | NP | 0 |
| **PGDM and GDM** | | | | | | | | | | | | | |
| Lv 2015 | Insulin analogs (GDM, DM 1/2) | Lispro vs Regular Ins or NPH | 0.65 | 0 (0-68) 0.90 | 4 | 2 | 1.25 | 0.42 | 1.25 | 0.42 | 1.54 | 0.64 | 15 |
| Lv 2015 | Insulin analogs (GDM, DM 1/2) | Lispro vs Regular Ins or NPH | 0.84 | 0 (0-61) 0.47 | 6 | 1 | 3.34 | NP | 3.34 | NP | 3.66 | NP | 7 |
| Pollex 2011 | Ins glargine/NPH (PGDM, GDM) | Glargine vs NPH | 0.96 | 0 (0-68) 0.76 | 5 | 0 | 0.2 | NP | 0.2 | NP | 0.21 | NP | 0 |
| Pollex 2011 | Ins glargine/NPH (PGDM, GDM) | Glargine vs NPH | 0.19 | 0 (0-73) 0.76 | 3 | 0 | 0.24 | NP | 0.24 | NP | 0.16 | NP | 0 |
| Pollex 2011 | Ins glargine/NPH (PGDM, GDM) | Glargine vs NPH | 0.57 | 19 (0-66) 0.28 | 7 | 0 | 0.36 | NP | 0.36 | NP | 0.49 | NP | 0 |

**Abbreviations:** GDM- Gestational diabetes mellitus; PGDM- Pregestational diabetes mellitus; DM 1/2- Diabetes mellitus type 1/2; sc-subcutaneous; inj- injections; Ins- Insulin; Mx- Management; NPH- Neutral Protamine Hagedorn; CSII- Continuous subcutaneous insulin infusion; MDI- Multiple daily injections

**Key:**

^α^ P-value from the Egger’s regression asymmetry test (P<0.10)

^β^ I^2^ metric of inconsistency (95% confidence interval) and the P-value of the Q test

^χ^ Observed number of statistically significant studies in each meta-analysis

^δ^ Expected number of statistically significant studies using the point estimate of each meta-analysis (from fixed effect, random effect of largest study accordingly) as the plausible effect size

^ε^P value of the excess statistical significance test

All statistical tests were two-sided
