# Supplementary material for: Diabetes and anti-diabetic interventions and the risk of gynaecological and obstetric morbidity: an umbrella review of the literature
Source: BMC Med. 2023 Apr 18;21:152. doi: 10.1186/s12916-023-02758-1 (PMC10114404; doi:10.1186/s12916-023-02758-1)
Supplement: Supplementary file 10 — Additional file 10: Table S7. Meta-analyses including only statistically significant* RCTs over the effect of any anti-diabetic intervention on the incidence of any obstetric or gynaecological disease. [file 12916_2023_2758_MOESM10_ESM.docx]

**Table S7. Meta-analyses including only statistically significant* RCTs over the effect of any anti-diabetic intervention on the incidence of any obstetric or gynaecological disease**

| **Author year** | **Intervention** | **Intervention contrast** | **Population** | **Outcome** | **Participants total** | **Events total** | **N of studies** | **Level of evidence (GRADE)** | **Random summary effect estimate (95% CI)** |
| --- | --- | --- | --- | --- | --- | --- | --- | --- | --- |
| Brown 2017 | Lifestyle interventions | ﻿Lifestyle intervention vs usual care/control | GDM | Shoulder dystocia | 2,894 | 57 | 5 | - | 0.37 (0.21-0.65) |
| Brown 2017 | Ins/Diet | Ins vs diet/standard care | GDM | Macrosomia | 717 | 70 | 3 | - | 0.30 (0.18-0.50) |
| Brown 2017 | Ins/Other Ins | NPH vs other Ins | GDM | Macrosomia | 84 | 9 | 2 | - | 0.10 (0.02-0.65) |
| Brown 2017 | Lifestyle interventions | ﻿Lifestyle intervention versus usual care/control | GDM | LGA | 2,994 | 450 | 6 | Moderate quality | 0.60 (0.50-0.72) |
| Brown 2017 | Lifestyle interventions | ﻿Lifestyle intervention versus usual care/control | GDM | Macrosomia | 3,422 | 499 | 7 | - | 0.64 (0.48-0.86) |
| Falavigna 2012 | GDM Tx/Usual antenatal care | GDM Tx vs usual antenatal care | GDM | Hypertensive disorders in pregnancy | 2,084 | 269 | 3 | Moderate quality | 0.64 (0.51-0.81) |
| Falavigna 2012 | GDM Tx/Usual antenatal care | GDM Tx vs usual antenatal care | GDM | LGA >90th centile | 2,242 | 356 | 4 | High quality | 0.57 (0.44-0.74) |
| Falavigna 2012 | GDM Tx/Usual antenatal care | GDM Tx vs usual antenatal care | GDM | Macrosomia (>4000g) | 3,157 | 412 | 6 | High quality | 0.47 (0.35-0.63) |
| Falavigna 2012 | GDM Tx/Usual antenatal care | GDM Tx vs usual antenatal care | GDM | Shoulder dystocia | 1,958 | 48 | 2 | Low quality | 0.41 (0.22-0.75) |
| Horvath 2010 | Tx for GDM | Intensive vs less intensive Tx | GDM | Shoulder dystocia | 775 | 20 | 6 | - | 0.31 (0.13-0.71) |
| Lau 2016 | Internet-Based Self-Monitoring | ﻿Internet-Based Self-Monitoring vs usual care | GDM, DM 1/2 | CS | 307 | 133 | 2 | - | 0.73 (0.56-0.97) |
| Tieu 2017 | Metformin/Ins | Metformin vs Ins | DM2, GDM | CS | 241 | 160 | 3 | Low quality | 0.75 (0.57-0.99) |
| Tieu 2017 | Metformin/Ins | Metformin vs Ins | DM2, GDM | Hyperbilirubinaemia | 220 | 40 | 2 | - | 0.45 (0.25-0.83) |
| Alwan 2009 | Treatment | Any specific Tx vs routine antenatal care | GDM | IOL | 1,068 | 374 | 2 | - | 1.33 (1.13-1.57) |
| Poolsup 2014 | Metformin/Ins | Metformin vs Ins | GDM | Gestational hypertension | 1,110 | 58 | 3 | - | 0.53 (0.31-0.91) |
| Hartling 2013 | Any Tx | Any Tx vs no Tx | GDM | Preeclampsia | 2,014 | 191 | 3 | - | 0.62 (0.43-0.89) |
| Hartling 2013 | Any Tx | Any Tx vs no Tx | GDM | Shoulder dystocia | 2040 | 51 | 3 | - | 0.42 (0.23-0.76) |
| Tufnell 2003 | Treatment | Any intensive Tx vs any minimal Tx | GDM/IGT | Neonatal hypoglycaemia | 194 | 17 | 2 |  | 0.25 (0.75-0.84) |
| Butalia 2017 | Metformin/Ins | Metformin vs Ins | GDM/DM2 | LGA | 1,549 | 315 | 7 | - | 0.80 (0.64-0.99) |
| Butalia 2017 | Metformin/Ins | Metformin vs Ins | GDM/DM2 | Neonatal hypoglycaemia | 2,120 | 296 | 14 | - | 0.62 (0.45-0.86) |
| Butalia 2017 | Metformin/Ins | Metformin vs Ins | GDM/DM2 | NICU admission | 1,822 | 360 | 10 | - | 0.72 (0.57-0.91) |
| Butalia 2017 | Metformin/Ins | Metformin vs Ins | GDM/DM2 | PIH | 1,160 | 87 | 4 | - | 0.56 (0.37-0.85) |
| Immanuel 2017 | Treated GDM in developed countries | Early onset vs late onset GDM (both treated) | GDM | NICU admission | 7,872 | 1,806 | 4 | Low quality | 1.13 (1.04-1.22) |
| Immanuel 2017 | Treated GDM in developed countries | Early onset vs late onset GDM (both treated) | GDM | Perinatal mortality | 9,010 | 41 | 6 | Low quality | 3.61 (1.90-6.84) |
| Kalafat 2018 | Metformin | Metformin vs Ins | GDM | Hypertensive disorders in pregnancy | 1,724 | 233 | 8 | Low quality | 0.71 (0.55-0.91) |
| Kalafat 2018 | Metformin | Metformin vs other drugs/placebo | GDM/ obesity | Hypertensive disorders in pregnancy | 3,120 | 376 | 15 | Very low quality | 0.75 (0.60-0.94) |
| Guo 2019 | Ins/Oral Tx | Metformin vs Ins | GDM | Preeclampsia | 3,402 | 289 | 14 | - | 0.67 (0.47-0.95) |
| Guo 2019 | Ins/Oral Tx | Metformin vs Ins | GDM | Neonatal hypoglycaemia | 2,755 | 581 | 15 | - | 0.57 (0.50-0.66) |
| Guo 2019 | Ins/Oral Tx | Glyburide vs Ins | GDM | Neonatal hypoglycaemia | 2,406 | 174 | 12 | - | 1.70 (1.11-2.59) |
| Guo 2019 | Ins/Oral Tx | Metformin vs Ins | GDM | Macrosomia >4kg | 2,331 | 272 | 13 | - | 0.70 (0.54-0.90) |
| Bao 2019 | Ins/Oral Tx | Metformin vs Ins | GDM | PIH | 1,526 | 105 | 5 | - | 0.64 (0.44-0.95) |
| Brown 2017c | Ins/Diet | Ins vs diet/standard care | GDM | Birth weight | 106 | 61 | 2 | - | (-)332.5 (-579.9 - -85.1) |
| Brown 2017a | Lifestyle interventions | ﻿Lifestyle intervention  vs usual care/control | GDM | Birth weight | 3,074 | 1,521 | 6 | - | (-)109.6 (-149.8 - -69.5) |
| Giuffrida 2003 | Diet/Ins | Diet alone vs diet/Ins combined | GDM | Macrosomia | 1,281 | 644 | 6 | - | (-)0.10 (-0.17 - -0.03) |
| Yamamoto 2018 | Dietary intervention | DASH vs control | GDM | Birth weight | 119 | 60 | 3 | Very low quality | (-)598.2 (-663.1 - -533.3) |
| Yamamoto 2018 | Dietary intervention | Soy protein vs control | GDM | Birth weight | 131 | 67 | 2 | Very low quality | (-)184.7 (-319.4 - -50.0) |
| Guo 2019 | Ins/Oral Tx | Ins vs Metformin | GDM | GA at delivery | 2295 | 1209 | 12 | - | 0.23 (0.12-0.34) |
| Tarry-Adkins 2019 | Ins/Oral Tx | Metformin vs Ins | GDM | Neonatal ponderal index | 986 | 488 | 3 | - | (-)0.13 (-0.26 - -0.002) |
| Tarry-Adkins 2019 | Ins/Oral Tx | Metformin vs Ins | GDM | Birth weight | 2,816 | 1,386 | 17 | - | (-)107.7 (-182.7 - -32.7) |
| Brown 2017c | Ins/Oral Tx | Ins vs Metformin | GDM | NICU admission | 2,306 | 445 | 10 | - | 1.45 (1.11-1.89) |

**Abbreviations:** RCT- Randomised controlled trial; Ins- Insulin; GDM- Gestational diabetes mellitus; NPH- Neutral protamine hagedorn; LGA- Large for gestational age; CS- Caesarean section; IOL- Induction of labour; Tx- Treatment; DM 1/2- Diabetes mellitus type 1/2; NICU- Neonatal intensive care unit; PIH- Pregnancy-induced hypertension; GA- Gestational age

**Key:**

* random p value< 0.05
