# Supplementary material for: Diabetes and anti-diabetic interventions and the risk of gynaecological and obstetric morbidity: an umbrella review of the literature
Source: BMC Med. 2023 Apr 18;21:152. doi: 10.1186/s12916-023-02758-1 (PMC10114404; doi:10.1186/s12916-023-02758-1)
Supplement: Supplementary file 11 — Additional file 11: Table S8. Risk of bias in the included interventional meta-analyses (RCTs). [file 12916_2023_2758_MOESM11_ESM.docx]

**Table S8. Risk of bias in the included interventional meta-analyses (RCTs)**

| **Author, year** | **Study type** | **Tool used** | **Subscale** | **Range** | **Results** | | |
| --- | --- | --- | --- | --- | --- | --- | --- |
| **Brown, 2017a** | | | | | **Low (-)** | **Unclear (?)** | **High (+)** |
|  | RCT | Cochrane Handbook for Systematic reviews of Interventions | Random sequence generation  Allocation concealment  Blinding of participants/personnel  Blinding of outcome  Incomplete outcome data  Selective reporting  Other bias | Low/Unclear/High risk | 6/10  4/10  4/10  5/10  7/10  3/10  9/10 | 4/10  6/10  1/10  5/10  1/10  1/10  0/10 | 0/10  0/10  5/10  0/10  2/10  6/10  1/10 |
| **Brown, 2017c** | | | | | **Low (-)** | **Unclear (?)** | **High (+)** |
|  | RCT | Cochrane Handbook for Systematic reviews of Interventions | Random sequence generation  Allocation concealment  Blinding of participants/personnel  Blinding of outcome  Incomplete outcome data  Selective reporting  Other bias | Low/Unclear/High risk | 1/5  0/5  0/5  0/5  4/5  0/5  1/5 | 3/5  4/5  1/5  5/5  0/5  1/5  1/5 | 1/5  1/5  4/5  0/5  1/5  4/5  3/5 |
| **Falavigna, 2012** | | | | | **Low** | **Uncertain** | **High** |
|  | RCT | Cochrane Handbook for Systematic reviews of Interventions | Random sequence generation  Allocation concealment  Blinding of participants/personnel  Incomplete outcome data | Low/Uncertain/High quality | 3/7  2/7  0/7  4/7 | 1/7  2/7  0/7  2/7 | 3/7  3/7  7/7  1/7 |
| **Horvath, 2010** | | | | | **Yes** | **Unclear** | **No** |
|  | RCT |  | Adequate randomisation  Adequate allocation concealment  Blinding of participants/caregivers  Blinding of end point assessment  ITT analyses | Yes/Unclear/No | 3/6  2/6  1/6  0/6  3/6 | 3/6  4/6  0/6  6/6  1/6 | 0/6  0/6  5/6  0/6  2/6 |
| **Overall** High potential for study bias | | | | | | | |
| **Lau, 2016** | | | | | **Low (-)** | **Unclear (?)** | **High (+)** |
|  | RCT | Cochrane Handbook Guidelines (2011) | Random sequence generation  Allocation concealment  Blinding of participants/personnel  Blinding of outcome  Incomplete outcome data  Selective reporting | Low/Unclear/High risk | 1/2  2/2  2/2  0/2  0/2  0/2 | 1/2  0/2  0/2  2/2  0/2  1/2 | 0/2  0/2  0/2  0/2  2/2  1/2 |
| **Tieu, 2017** | | | | |  |  |  |
|  | RCT | Cochrane Handbook Guidelines (2011) | Random sequence generation  Allocation concealment  Blinding of participants/personnel  Blinding of outcome  Incomplete outcome data  Selective reporting  Other bias | Low/Unclear/High risk | 1/3  1/3  3/3  0/3  1/3  0/3  0/3 | 0/3  0/3  0/3  3/3  1/3  2/3  3/3 | 2/3  2/3  0/3  0/3  1/3  1/3  0/3 |
| **Alwan. 2009** | | | | | **Low (-)** | **Unclear (?)** | **High (+)** |
|  | RCT | Cochrane Handbook Guidelines (2011) | Allocation concealment | Low/Unclear/High risk | 2/2 | 0/2 | 0/2 |
| **Poolsup, 2014** | | | | | **Low (-)** | **Unclear (?)** | **High (+)** |
|  | RCT | Cochrane Handbook Guidelines (2011) | Random sequence generation  Allocation concealment  Blinding of participants/personnel  Blinding of outcome  Incomplete outcome data  Selective reporting  Other bias | Low/Unclear/High risk | 0/3  0/3  3/3  2/3  0/3  0/3  0/3 | 1/3  2/3  0/3  0/3  0/3  0/3  0/3 | 2/3  1/3  0/3  1/3  3/3  3/3  3/3 |
| **Hartling, 2013** | | | | |  |  |  |
|  | RCT |  | Risk of bias  Consistency  Directness  Precision  Overall strength of evidence |  | **Preeclampsia/Shoulder dystocia**  Low/Medium  Consistent/Consistent  Direct/Direct  Imprecise/Precise  Moderate/Moderate (favours treatment) | | |
| **Tufnell, 2003** | | | | | **Low (-)** | **Unclear (?)** | **High (+)** |
|  | RCT | Cochrane Reviewer’s Handbook (Clarke 2000) | Allocation concealment |  | 1/2 | 1/2 | 0/2 |
| **Butalia, 2017** | | | | | **Yes** | **Unclear** | **No** |
|  | RCT | Risk of bias assessment tool recommended by the Cochrane Neonatal Review Group | Allocation concealment  Selection criteria (inclusion/exclusion)  Group comparability  Assessors blinded  ITT  Loss to follow-up reported | Yes/Unclear/No | 8/15  14/15  10/15  3/15  10/15  14/15 | 6/15  0/15  1/15  1/15  1/15  0/15 | 1/15  1/15  14/15  11/15  4/15  1/15 |
| **Kalafat, 2018** | | | | | **Low (-)** | **Unclear (?)** | **High (+)** |
|  | RCT | Cochrane risk of bias tool | Random sequence generation  Allocation concealment  Blinding of participants/personnel  Blinding of outcome  Incomplete outcome data  Selective reporting  Other bias | Low/Unclear/High risk | 10/15  7/15  15/15  15/15  13/15  9/15  5/15 | 4/15  7/15  0/15  0/15  1/15  3/15  10/15 | 1/15  1/15  0/15  0/15  1/15  3/15  0/15 |
| **Guo, 2019** | | | | | **Low (-)** | **Unclear (?)** | **High (+)** |
|  | RCT | Cochrane Handbook for Systematic reviews of Interventions | Random sequence generation  Allocation concealment  Blinding of participants/personnel  Blinding of outcome  Incomplete outcome data  Selective reporting  Other bias | Low/Unclear/High risk | 2/32  0/32  0/32  0/32  0/32  0/32  0/32 | 19/32  21/32  23/32  22/32  1/32  16/32  32/32 | 11/32  11/32  9/32  10/32  31/32  16/32  0/32 |
| **Bao, 2019** | | | | | **Low (-)** | **Unclear (?)** | **High (+)** |
|  | RCT | Cochrane Handbook for Systematic reviews of Interventions | Random sequence generation  Allocation concealment  Blinding of participants/personnel  Blinding of outcome  Incomplete outcome data  Selective reporting  Other bias | Low/Unclear/High risk | 1/5  0/5  0/5  0/5  0/5  0/5  0/5 | 0/5  0/5  5/5  5/5  0/5  0/5  0/5 | 4/5  5/5  0/5  0/5  5/5  5/5  5/5 |
| **Yamamoto, 2018** | | | | | **Low (-)** | **Unclear (?)** | **High (+)** |
|  | RCT | Cochrane Collaboration tool | Random sequence generation  Allocation concealment  Blinding of participants/personnel  Blinding of outcome  Incomplete outcome data  Selective reporting  Other bias | Low/Unclear/High risk | 0/5  0/5  1/5  2/5  2/5  2/5  3/5 | 1/5  5/5  3/5  2/5  2/5  3/5  0/5 | 4/5  0/5  1/5  1/5  1/5  0/5  2/5 |
| **Tarry-Adkins, 2019** | | | | | **Low (-)** | **Unclear (?)** | **High (+)** |
|  | RCT | Cochrane Collaboration tool | Random sequence generation  Allocation concealment  Blinding of participants/personnel  Blinding of outcome  Incomplete outcome data  Selective reporting  Other bias | Low/Unclear/High risk | 3/17  0/17  17/17  3/17  5/17  0/17  1/17 | 2/17  12/17  0/17  11/17  1/17  1/17  1/17 | 12/17  5/17  0/17  3/17  11/17  16/17  15/17 |
